# Molecular Engineering Empowers Phenanthraquinone Organic Cathodes with Exceptional Cycling Stability for Lithium‐ and Aqueous Zinc‐Ion Batteries

**DOI:** 10.1002/advs.202506749

**Published:** 2025-09-11

**Authors:** Susu Li, Haoyu Zhang, Jixing Yang, Yunhua Xu, Yuesheng Li

**Affiliations:** ^1^ School of Materials Science and Engineering Tianjin Key Laboratory of Composite and Functional Materials State Key Laboratory of Advanced Materials for Intelligent Sensing Tianjin University Tianjin 300072 China

**Keywords:** aqueous zinc‐ion batteries, design and synthesis, lithium‐ion batteries, organic cathode materials, phenanthraquinone

## Abstract

Organic electrode materials have garnered great attention in recent years, owing to their resource sustainability, structural diversity, and superior compatibility with various ionic species. Among them, quinone‐based compounds have attracted particular interest. Notably, compared with para‐quinone analogs (e.g., anthraquinone), ortho‐quinone‐based electrode materials (e.g., phenanthraquinone) demonstrate higher redox potential, which contributes to achieving higher energy density in battery applications. However, their practical applications have been limited by severe solubility in electrolytes, resulting in poor cycle performance. To improve it, two novel phenanthraquinone‐derived molecules, 1,4‐bis(9,10‐phenanthraquinonyl)benzene (BPQB) and 1,3,5‐tri(9,10‐phenanthraquinonyl)benzene (TPQB), are rationally designed, synthesized, and applied as cathode materials for both lithium‐ion batteries (LIBs) and aqueous zinc‐ion batteries (AZIBs). Comprehensive electrochemical evaluations reveal remarkable cycling stability and rate performance of TPQB. The TPQB cathodes for LIBs achieve a high‐capacity retention of 76.8% after 1,000 cycles at 5 C, while exhibiting extraordinary ultra‐long cycle life in AZIBs with 93.2% capacity retention over 6,000 cycles at 5 C. Their charge storage mechanisms are elucidated through various characterization methods. The work presents a novel molecular engineering strategy that effectively inhibits the dissolution of phenanthraquinone‐derived electrode materials and thus realizes excellent electrochemical performance across diverse battery systems.

## Graphical abstract

Two new phenanthraquinone‐based molecules are rationally designed, synthesized, and employed as dual metal‐ion battery cathode materials. The ingenious molecular structure design effectively enhances intermolecular interactions and reduces solubility, demonstrating superb long‐term cycling stability for both lithium‐ion (76.8% capacity retention after 1000 cycles at 5 C) and aqueous zinc‐ion batteries (93.2% capacity retention after 6000 cycles at 5 C).

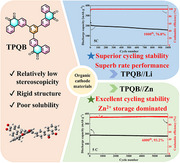

## 1 Introduction

Lithium‐ion batteries (LIBs) have been booming in recent years, primarily driven by their vital role in portable electronics, electric vehicles, and grid‐scale energy storage systems.^[^ ^1^, ^2^, ^3^ ^]^ Currently, commercial inorganic cathode materials, such as LiCoO_2_ and LiNi_0.6_Co_0.2_Mn_0.2_O_2_,^[^ ^4^, ^5^ ^]^ rely on scarce transition metal resources, which are faced with challenges of unsustainability and escalating cost, especially with the further development of LIBs in the foreseeable future.^[^ ^6^, ^7^ ^]^ In contrast, organic cathode materials (OCMs), mainly composed of abundant C, H, O, and N elements, have significant advantages in resource availability and sustainability.^[^ ^8^, ^9^, ^10^ ^]^ Additionally, the inherent structural diversity and tunability of OCMs are capable of enhancing or tuning their electrochemical performance, such as voltage, capacity, cycle performance, and rate capability.^[^ ^11^, ^12^ ^]^ In particular, organic electrode materials exhibit enhanced adaptability to diverse ion sizes because of their expanded intermolecular spaces, while inorganic materials with rigid crystal structures are typically limited to storing only one type of metal ion.^[^ ^13^ ^]^ This unique feature endows OCMs with simultaneous accommodation capabilities for both monovalent (e.g., Li^+^, Na^+^, K^+^) and multivalent cations (e.g., Zn^2+^, Mg^2+^, Al^3+^).^[^ ^14^ ^]^ Among these multivalent metal‐organic battery systems, zinc‐organic batteries stand out by combining the intrinsic nonflammability of aqueous electrolytes with the economic advantages of zinc metal (high abundance and low cost), making them highly suitable for grid‐scale energy storage platforms.^[^ ^15^, ^16^ ^]^ Overall, owing to all the above‐mentioned merits, OCMs‐based organic batteries have become one of the most promising candidates for next‐generation rechargeable battery technologies.^[^ ^17^ ^]^

Among the studied OCMs, because of their good chemical stability, superior redox reversibility, and high specific capacity, conjugated carbonyl compounds have received great attention, most typically, quinones, such as 1,4‐benzoquinone (BQ), 1,4‐naphthoquinone (NQ), 9,10‐anthraquinone (AQ), and 9,10‐phenanthraquinone (PQ).^[^ ^18^, ^19^, ^20^, ^21^ ^]^ Compared with BQ and NQ, AQ has a larger and more stable conjugated system and more sites for structural modification. However, its discharge voltage is relatively low (ca. 2.3 V vs Li/Li^+^).^[^ ^22^ ^]^ As the isomer of AQ, PQ can achieve high discharge voltage (ca. 2.6 V vs Li/Li^+^) owing to the proximity of the two electron‐withdrawing carbonyl groups, which can obtain higher energy density.^[^ ^23^, ^24^ ^]^ Moreover, PQ is more conducive to coordinating with zinc ions because of the coordination chelation of ortho‐dicarbonyl with divalent metal ions.^[^ ^25^, ^26^ ^]^ However, PQ and its discharged intermediates dissolve very easily in liquid electrolytes (both organic and aqueous), leading to significant capacity loss over time and severely limiting their practical applications.^[^ ^27^, ^28^ ^]^

In fact, the dissolution issue is indeed one of the pivotal challenges that currently hinder the advancement of OCMs.^[^ ^11^ ^]^ To tackle it, many strategies have been explored.^[^ ^29^ ^]^ Among these, chemical modification of molecular structure has been proven to be one of the most effective methods to reduce solubility by enhancing intermolecular interaction, including introducing strong polar groups^[^ ^24^, ^30^ ^]^ and expanding the planar π‐conjugated system.^[^ ^31^, ^32^ ^]^ Note that, although some reported materials exhibit low inherent dissolution propensity, progressive capacity decay is still often observed during cycling, primarily attributed to the enhanced solubilities of their discharged species.^[^ ^33^, ^34^ ^]^ As batteries are integrated systems composed of multiple components, beyond low‐solubility molecular design, key components such as separator and electrolyte, which have direct contact with the cathode, can also significantly impact the electrochemical performance of OCMs and thus require synergistic design.^[^ ^35^, ^36^, ^37^, ^38^ ^]^ Recently, our group has developed an in situ‐formed gel polymer electrolyte initiated by Nafion‐coated separator via cationic ring‐opening polymerization of 1,3‐dioxolane (DOL), a common solvent for electrolytes.^[^ ^39^ ^]^ This engineered gel electrolyte/functional separator system can effectively inhibit the shuttle of soluble discharged products of carbonyl‐based electrode materials to the anode side through charge repulsion of functionalized separator and physical confinement provided by gel polymer electrolyte, realizing greatly enhanced cycling stability for LIBs. Furthermore, active molecules with a larger size have been shown to be more effectively shielded. Therefore, it is anticipated that combining rational molecular structure design with our novel gel/separator system can synergistically improve the cycling stability of PQ‐based electrode materials for LIBs.

In order to achieve high cycle performance through the aforementioned gel/separator system, it is necessary to synthesize an insoluble PQ‐derived molecule with a large molecular size. The most effective method to reduce the solubility of conjugated structural units such as PQ is to expand its conjugated structure, enhancing the intermolecular *π*‐*π* interaction.^[^ ^29^, ^40^ ^]^ Nonetheless, when the expansion of the conjugated system is insufficient, the reduction in solubility is limited;^[^ ^41^ ^]^ an excessively large conjugated system may cause some side effects, such as reduced specific capacity, lower voltage, and difficulties in synthesis.^[^ ^41^, ^42^, ^43^ ^]^ Based on the above analyses, it is proposed that constructing rigid molecules with minimal steric hindrance, whose conjugated structures are connected exclusively by C─C single bond, can effectively reduce the solubility of PQ‐based materials. For example, bonding two or three PQ units to the 1,4‐ or 1,3,5‐positions of a benzene ring not only largely increases the molecular size but also reduces steric hindrance, thereby minimizing overall stereoscopicity. Due to this rigid and low‐steric structure, π‐π interaction would still exist between conjugated units of different molecules. Although these interactions are not as strong as those in fused‐ring conjugated planar structures of the same size, the cumulative effect of multiple conjugated units can similarly enhance intermolecular interaction. Moreover, the low molecular weight of the non‐active benzene moiety, combined with its distribution across two or three redox‐active PQ units per molecule, results in minimal specific capacity degradation.

To validate the above concept, herein, 1,4‐bis(9,10‐phenanthraquinonyl)benzene (BPQB) and 1,3,5‐tri(9,10‐phenanthraquinonyl)benzene (TPQB) were synthesized and applied as cathode materials for both LIBs and aqueous zinc‐ion batteries (AZIBs). It is found that both solubilities of BPQB and TPQB are significantly reduced. When the gel electrolyte/functional separator system was employed in LIB system, both BPQB and TPQB electrodes exhibited commendable electrochemical performance; however, TPQB demonstrates superior characteristics with higher active site utilization and better cycling stability in a comparative evaluation. The TPQB cathode displays a high capacity of 229.8 mAh g^−1^ at 0.2 C and can stably cycle for 1000 times with a high‐capacity retention of 76.8%. The electrochemical performance of TPQB as a cathode material for AZIBs was studied using a conventional aqueous electrolyte. Thanks to the successful molecular design, the AZIBs based on this TPQB cathode material also exhibit exceptional electrochemical performance without any modification to other battery components. Remarkably, it achieves a high capacity of 225.8 mAh g^−1^ at 0.1 C and outstanding capacity retention of 93.2% after ultralong cycles of 6000 times at 5 C, demonstrating its great potential for long‐term energy storage applications. To the best of our knowledge, the cycling stability demonstrated by TPQB‐based cathodes, whether in LIBs or AZIBs, represents the highest performance reported to date among all PQ‐derived small molecule electrode materials. This work not only demonstrates the effectiveness of our molecular design but also further establishes the unique capability of organic electrodes in reversibly accommodating diverse metal ions (e.g., Li⁺, Zn^2^⁺), offering a promising pathway for next‐generation low‐cost battery technologies.

## 2 Results and Discussion

### 2.1 Molecular Synthesis and Characterization

**Figure 1:**
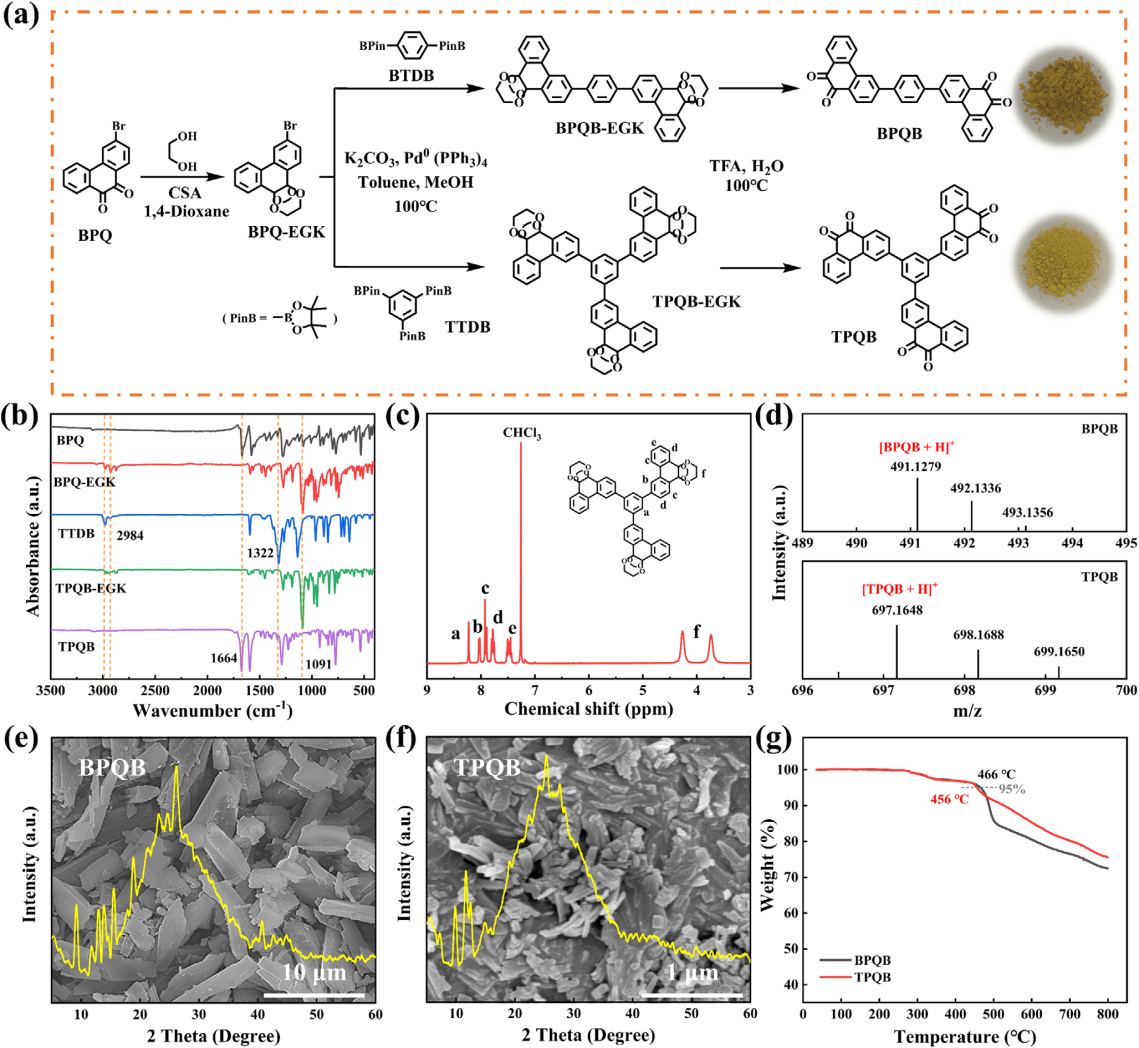
Synthesis routes and structure characterization results. (a) Synthesis routes of BPQB and TPQB. (b) FTIR spectra of the raw materials, intermediate products, and TPQB. (c) ^1^H NMR spectra of TPQB‐EGK. (d) MS spectra of BPQB and TPQB. XRD patterns and SEM images of the (e) BPQB and (f) TPQB. (g) TGA curves (N_2_, 10 °C min^−1^).

The detailed synthesis routes of target molecules, BPQB and TPQB, are illustrated in **Figure** **1**a. Notably, the PQ is oxidizing,^[^ ^44^ ^]^ which is prone to induce undesired side reactions with palladium catalyst during the Suzuki coupling reaction. To avoid this issue, pre‐protection of PQ's ortho‐dicarbonyl group with ethylene glycol (EG) is indispensable.^[^ ^45^ ^]^ Subsequent deprotection using trifluoroacetic acid (TFA)/H_2_O can efficiently regenerate carbonyl groups, yielding the desired molecules. Due to the structural analogy between BPQB and TPQB, the latter is chosen as a representative molecule to thoroughly analyze. Initial structural validation of 3‐bromophenanthrene‐9,10‐di(ethyleneglycol)ketal (BPQ‐EGK) was achieved by the ^1^H nuclear magnetic resonance (NMR) spectroscopy (Figure S1, Supporting Information). In the Fourier transform infrared (FTIR) spectroscopy (Figure 1b), protection of the ortho‐dicarbonyl group is further verified by the disappearance of the characteristic peak for carbonyl groups (1664 cm^−1^) concurrently with the emergence of new peaks for cyclic ketal structure (1091 cm^−1^ (C─O─C) and 2980 cm^−1^ (─CH_2_─)). The protected intermediate, TPQB‐EGK, was then synthesized by Pd^0^(PPh_3_)_4_‐catalyzed Suzuki coupling reaction. Its FTIR spectrum displays the presence of peaks for cyclic ketal and the absence of two characteristic peaks at 2984 cm^−1^ (C─H) and 1322 cm^−1^ (B─O) for 1,3,5‐tris(4,4,5,5‐tetramethyl‐1,3,2‐dioxaborolan‐2‐yl)benzene (TTDB), implying the successful synthesis of TPQB‐EGK (Figure 1b). The structure of TPQB‐EGK was further proved by the ^1^H NMR spectrum, as shown in Figure 1c, whose chemical shifts and area ratios of the peaks are in excellent agreement with its expected chemical structure. The successful synthesis of the objective product, TPQB, was initially verified by FTIR spectroscopy. As shown in Figure 1b, the characteristic peaks associated with the cyclic ketal structure are completely invisible, while the carbonyl peak at 1664 cm^−1^ is renewedly detected, thereby verifying the formation of the target molecule. The ^1^H NMR spectra of BPQB‐EGK and FTIR spectra of BPQB were obtained under identical experimental conditions (Figures S2 and S3, Supporting Information). Since the very poor solubilities of BPQB and TPQB in deuterated solvents such as dimethyl sulfoxide (DMSO‐*d6*), chloroform (CDCl_3_), and CF_3_CO_2_D, their ^1^H NMR spectra can not be obtained. Mass spectrum (MS) was employed to further verify the definitive molecular validation (Figure 1d). The measured characteristic m/z values are 491.1279 and 697.1648, respectively, which are almost the same as their theoretical values of 491.1205 and 697.1573 for the [BPQB+H]^+^ and [TPQB+H]^+^ ions. Moreover, the solid‐state ^13^C NMR spectra of BPQB and TPQB were also tested to prove molecular structures (Figure S4, Supporting Information). All the above results collectively demonstrate the successful syntheses of BPQB and TPQB.

Crystallinity, powder morphology, and thermal stability of the newly obtained BPQB and TPQB were explored by X‐ray diffraction (XRD) pattern, scanning electron microscopy (SEM), and thermogravimetric analysis (TGA), respectively. The XRD patterns (Figure 1e,f) reveal incomplete crystallization states for BPQB and TPQB, characterized by broad diffraction peaks superimposed with low‐intensity sharp peaks. This phenomenon likely originates from direct precipitation during the reaction process, which fails to provide the necessary thermodynamic conditions for ordered crystal growth. Their powder morphologies were examined using SEM, as shown in Figure 1e,f, showing different architectural features, with BPQB exhibiting irregular sheets and TPQB presenting as thick rods. Note that the particle size of BPQB is obviously larger than that of TPQB, which may affect their electrochemical performance. The uniform distributions of C and O elements in BPQB and TPQB powders are illustrated by the TEM–energy‐dispersive spectroscopy (EDS) mapping images (Figure S5, Supporting Information). As depicted in Figure 1g, the 5% weight loss temperatures of both BPQB and TPQB samples are ≈460 °C, indicating high thermal stability, which is beneficial to achieve good safety and reliability in battery systems.

### 2.2 Theoretical Calculation and Solubility Comparison

**Figure 2:**
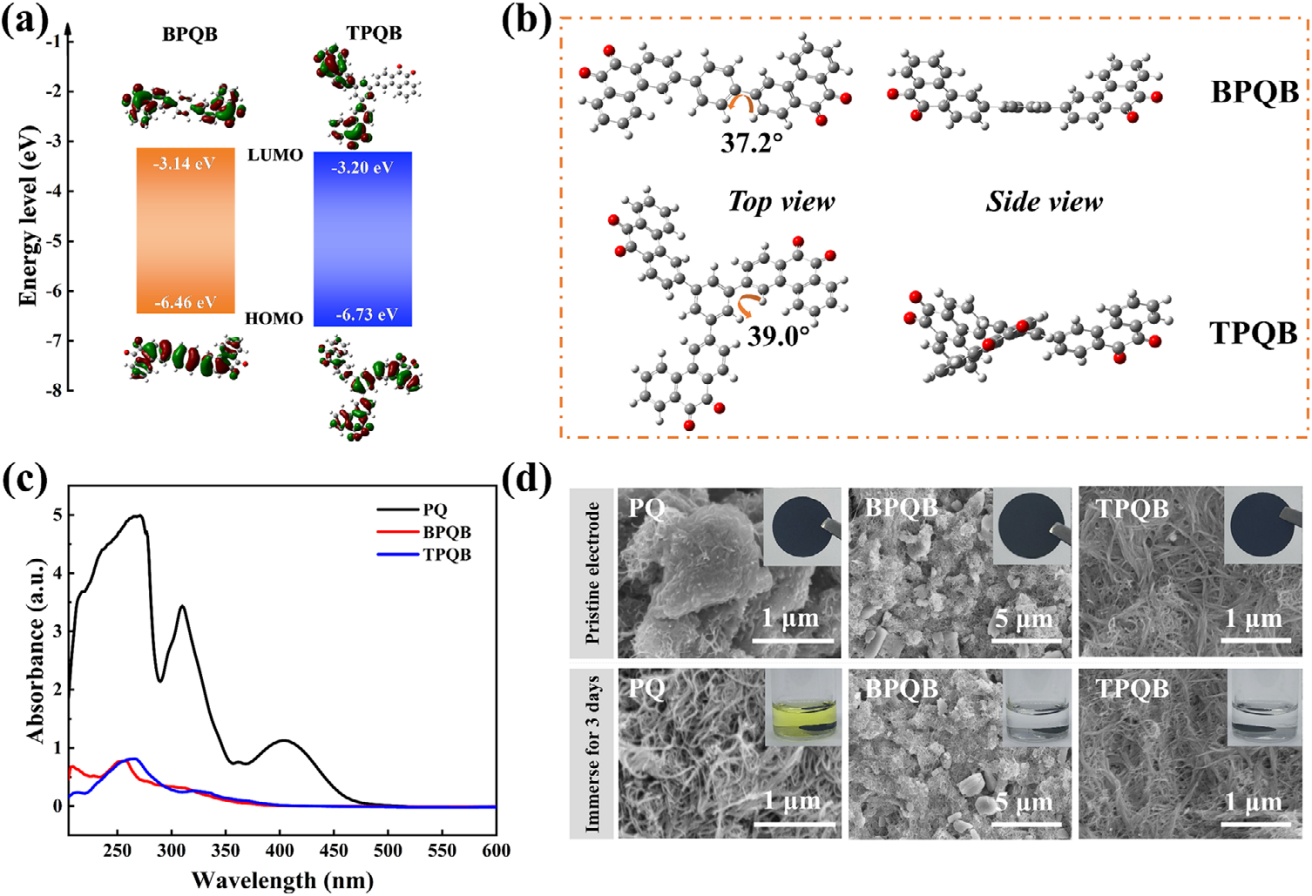
Theoretical calculation and solubility comparison. (a) Calculated LUMO/HOMO energy levels of BPQB and TPQB. (b) Optimized geometries of BPQB and TPQB by DFT calculations viewed from top and side, and their corresponding dihedral angles (C: dark gray; H: light gray; O: red). (c) UV–vis spectra of the saturated solution for PQ, BPQB, and TPQB. (d) SEM images of the pristine electrodes and soaked electrodes in electrolyte for 3 days. The inset in each SEM image is the photo of its corresponding pristine electrodes or the soaking solutions of electrodes.

Density functional theory (DFT) calculations were carried out to investigate molecular and electronic structures of the two new PQ‐derivatives. **Figure** **2**a depicts the frontier molecular orbitals of BPQB and TPQB. The lowest unoccupied molecular orbital (LUMO) levels of BPQB and TPQB are found to be −3.12 and −3.20 eV, respectively. Generally, a lower LUMO energy level corresponds to a higher reduction potential.^[^ ^29^, ^46^, ^47^ ^]^ Compared with AQ‐based analogs (both are −2.90 eV, Figure S6, Supporting Information), BPQB and TPQB exhibit relatively lower LUMO levels, which is well in accord with the expected higher discharge voltage of ortho‐quinones. Figure 2b displays the optimized molecular structures of BPQB and TPQB from different observation angles. Obviously, both molecules demonstrate relatively low stereoscopicity, which is attributed to the minimal steric hindrance between conjugated units within the molecules. Our previous studies found that dihedral angles below 40° between conjugated units in rigid molecules promote coplanarity, enhancing conjugation and intermolecular interactions (e.g., *π*‐*π* stacking), thereby reducing solubility.^[^ ^48^, ^49^ ^]^ The intramolecular dihedral angles are 37.2° and 39.0° for BPQB and TPQB, respectively, suggesting poor solubilities. Besides, uniform electronic distributions of the highest occupied molecular orbitals (HOMO) levels of BPQB and TPQB (Figure 2a) also validate the presence of conjugation interaction between PQ units and benzene because of their low dihedral angles.

Solubility reduction of BPQB and TPQB relative to PQ was experimentally proved through systematic comparative analyses. First, 15 mg of each powder was added into 1 mL of mixed solvents (DOL/1,2‐dimethoxyethane (DME), V/V = 1/1), followed by stirring for 3 days. As shown in Figure S7 (Supporting Information), the PQ powders dissolve completely, whereas the soaking solutions of BPQB and TPQB still exhibit many visible precipitates, providing preliminary evidence of reduced solubility. To further compare their solubilities, the supernatants of three compounds were tested by the UV–vis spectroscopic analysis. As shown in Figure 2c, absorbance values of the immersed solvents for BPQB and TPQB are significantly lower than that of PQ, which confirms the reduced solubility. Furthermore, the electrodes of PQ, BPQB, and TPQB were immersed in DOL/DME mixed solvent for 3 days, and the color change of the solutions was recorded (insets in Figure 2d). The solution after soaking PQ electrode exhibits a distinct yellow color, while those with the BPQB and TPQB electrodes remain almost colorless and transparent. Surface morphologies of these electrodes (pristine and immersed for 3 days) were further examined using SEM. As shown in Figure 2d, after immersion, the active materials of PQ electrode entirely dissolve, while the BPQB and TPQB electrodes show no significant changes. These results clearly demonstrate that our molecular engineering strategy successfully reduces the solubilities of BPQB and TPQB, highlighting their potential for enhanced cycle performance.

### 2.3 Electrochemical Performance of BPQB and TPQB Cathodes for LIBs

The electrochemical performance of BPQB and TPQB cathodes was evaluated in coin‐type cells with lithium metal as the anode, where 2 m lithium bis(trifluoromethanesulfonyl)imide (LiTFSI) and 0.6 m poly(ethylene oxide) (PEO) in DOL/DME (V/V = 1/1) serve as the electrolyte system. Nafion‐coated polypropylene (PP) separator was employed in the battery. The sulfonic acid groups (─SO_3_H) of Nafion polymer can induce cationic ring‐opening polymerization of DOL inside of batteries, in situ forming gel polymer electrolyte (Figures S8 and S9, Supporting Information).^[^ ^39^ ^]^

**Figure 3:**
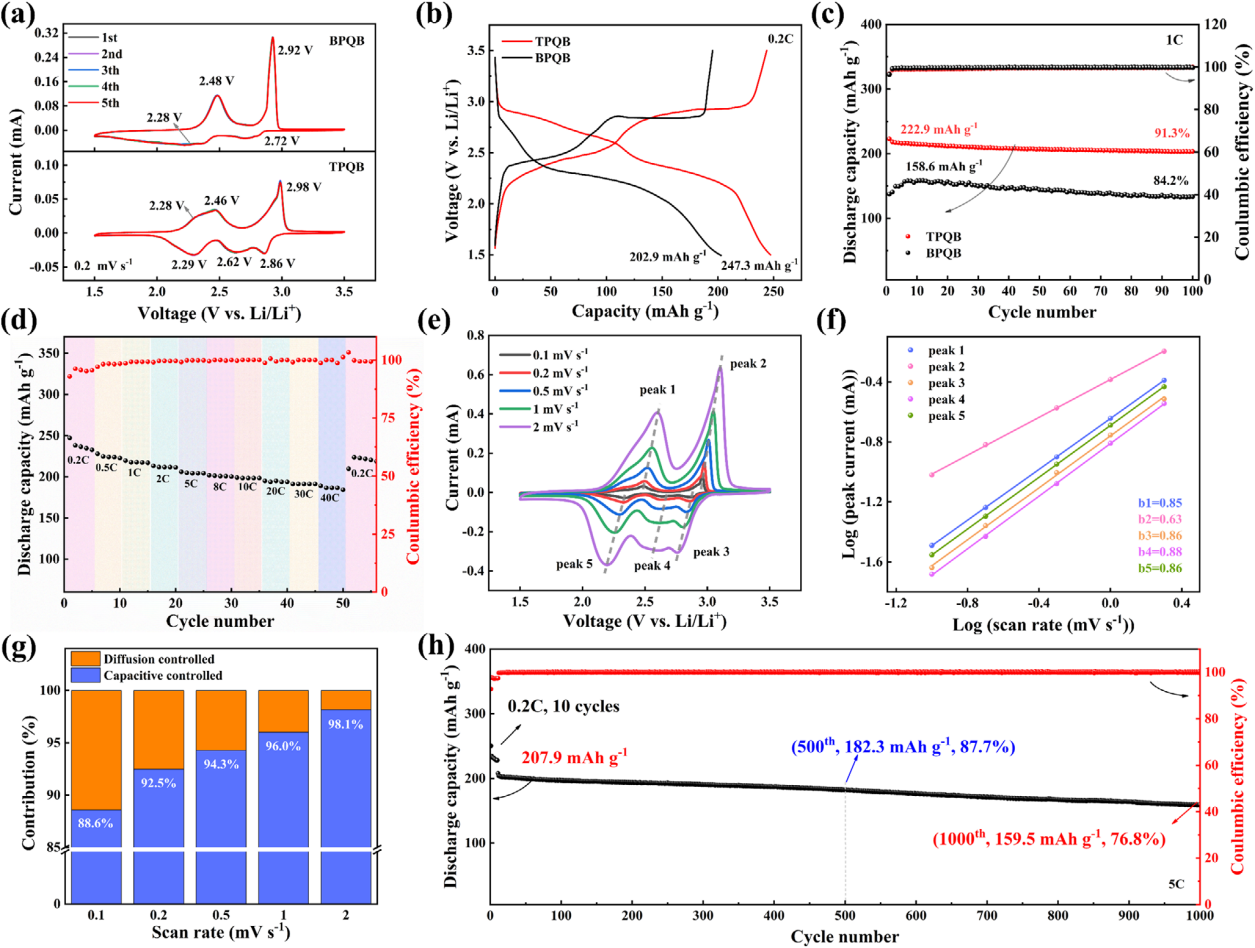
Electrochemical performance of BPQB and TPQB cathodes in LIBs. (a) CV curves at a scan rate of 0.2 mV s^−1^. (b) GCD profiles of their first cycles at 0.2 C. (c) Cycle performance at 1 C. (d) Rate performance of TPQB electrode at different current densities. (e) CV curves of TPQB electrode at different scan rates and (f) the corresponding plots of log(*i*, peak current) versus log(*v*, scan rate) for CV curves at different scan rates. (g) Capacity contribution ratios of diffusion control (blue) and surface control (orange) at different scan rates, and (h) long‐term cycle performance at 5 C of TPQB electrode.

The redox properties of BPQB and TPQB electrodes were first investigated by cyclic voltammetry (CV) within an operational voltage window of 1.5–3.5 V (vs Li/Li^+^) at 0.2 mV s^−1^. As shown in **Figure** **3**a, the CV curves of BPQB electrode exhibit two pairs of redox peaks centered at 2.28 V/2.48 V and 2.72 V/2.92 V, corresponding to the four‐electron reaction of BPQB. The CV curves of TPQB electrode demonstrate three pairs of redox peaks at 2.28 V/2.29 V, 2.46 V/2.62 V, and 2.86 V/2.98 V, which are related to the six‐electron reaction of TPQB. Notably, both BPQB and TPQB maintain invariant redox peak current and potential during cycling, indicating good chemical stability and excellent electrochemical reversibility. To investigate the electrochemical performance of BPQB and TPQB cathodes, galvanostatic charge/discharge (GCD) measurements were carried out at 0.2 C (1 C for BPQB and TPQB is 218.7 and 231.0 mA g^−1^, respectively). Figure 3b presents the first‐cycle GCD profiles of both electrodes, revealing multi‐platform charging and discharging behavior, which are consistent with their CV curves. Notably, after deducting the capacity of 17.5 mAh g^−1^ from graphene contribution (Figure S10, Supporting Information), TPQB cathode achieves an exceptional initial discharge capacity of 229.8 mAh g^−1^. This value approaches its theoretical specific capacity (231.0 mAh g^−1^), demonstrating that the redox active site utilization rate is ≈100%. In contrast, BPQB cathode delivers relatively poor capacity expression (180.4 mAh g^−1^ after removing the contribution of carbon), which is lower than its theoretical value (218.7 mAh g^−1^). This discrepancy is possibly attributed to the larger particle size of BPQB, which has been previously observed in SEM images (Figures 1e,f, and 2d). Moreover, BPQB exhibits a lower discharge voltage plateau compared to TPQB, which can be attributed to structural differences. The three PQ units connect with the central benzene ring (an electron‐donating moiety) in TPQB resulting in a smaller voltage reduction than that in BPQB with only two PQ units.^[^ ^49^ ^]^ This trend is also supported by DFT calculations (Figure 2a), indicating that TPQB exhibits a lower LUMO energy level compared to BPQB.

Cycle performance and Coulombic efficiencies (CEs) of BPQB and TPQB cathodes at 1 C were exhibited in Figure 3c. Following initial activation cycles, both batteries achieve exceptional CEs stabilized ≈100%, highlighting the poor solubility and thus structural stability of molecules. Both BPQB and TPQB cathodes display quite good cycle performance. After cycling for 100 times at 1 C, BPQB retains 84.2% of its initial capacity, while TPQB shows superior capacity retention of 90.7%. This performance divergence mainly originates from the difference of molecular size. Our previous study has demonstrated that the engineered gel electrolyte/functional separator system is more effective in shielding large‐sized active molecules and thus improving the cycling stability of TPQB cathode for LIBs.^[^ ^39^ ^]^ To further validate the dual strategies of molecular engineering (BPQB/TPQB) and gel electrolyte/functional separator system for mitigating capacity fade, the cycle performance of the PQ cathode in gel electrolyte/functional separator system and BPQB/TPQB cathode in the liquid electrolyte were also tested, respectively (Figure S11, Supporting Information). Given these combined advantages in both higher capacity expression and cycling stability, TPQB was selected for subsequent in‐depth electrochemical characterizations.

The rate performance of TPQB cathode was then evaluated under different current densities from 0.2 C to 40 C in the voltage window of 1.5–3.5 V (Figure 3d). Impressively, TPQB cathode displays outstanding high‐rate capability. It exhibits discharge capacities of 236.7, 228.0, 217.8, 211.8, 204.3, 200.9, 198.6, 195.0, 191.3, and 186.7 mAh g^−1^ at 0.2, 0.5, 1, 2, 5, 8, 10, 20, 30, and 40 C, respectively. As the current density increases from 0.2 C to 10 C, high‐capacity retention of 83.9% is achieved. Even at a very high current density of 40 C, the TPQB cathode still sustains a high‐capacity retention of 78.9%. Remarkably, when the current density returns from 40 C to 0.2 C, the TPQB cathode regains a capacity of 223.6 mAh g^−1^ (94.5% of initial capacity), further proving its superb redox reversibility and stability. The GCD profiles of TPQB cathodes at various current densities reveal an unobvious polarization phenomenon with the increasing current density (Figure S12, Supporting Information), implying good reaction kinetics. Moreover, to further validate the excellent rate performance, the galvanostatic intermittent titration technique (GITT) was performed (Figure S13, Supporting Information). The Li^+^ diffusion coefficients (*D*) of TPQB cathode were estimated based on established methodologies reported in the literature.^[^ ^50^ ^]^ It is found that the calculated *D* values are among 0.3 × 10^−10^ to 0.1 × 10^−12^ cm^2^ s^−1^, suggesting rapid reaction kinetics.

Electrochemical reaction kinetics of the TPQB cathode were comprehensively investigated through CV measurement at various scan rates from 0.1 to 2 mV s^−1^ (Figure 3e). Remarkably, even at high scanning rates, the CV curves have no apparent shape deformation except subtle shifts caused by polarization, which supports the exceptional rate performance and fast reaction kinetics of TPQB electrode. In general, the total capacity consists of the faradaic process and the capacitive process. As shown in Figure 3f, a quantitative analysis of capacity contribution was performed using the power‐law relationship between peak current (*i*) and scan rate (*v*). According to the formula *i* = *a v^b^*,^[^ ^51^ ^]^ the *b* values are fitted to be 0.85, 0.63, 0.86, 0.88, and 0.86 for the five cathodic or anodic peaks (peak 1–5, Figure 3e), respectively. Proverbially, the *b* value approaching to 0.5 represents a diffusion‐controlled process; instead, if the *b* is close to 1, the electrochemical behavior relies on the capacitance process. Therefore, it is suggested that the capacity of TPQB cathode is controlled by both diffusion and pseudocapacitive behaviors. The *b* value for peak 2 is relatively lower than those of others, which may arise from partial peak overlap during the oxidative process.^[^ ^52^ ^]^ The quantitative capacity contribution at different scan rates is calculated by the formula *i* = *k_1_ v* + *k_2_ v* ^1/2^.^[^ ^53^ ^]^ As shown in Figure 3g and Figure S14 (Supporting Information), with the scanning rate increases, the capacitive control contribution gradually increases from 88.2% to 98.1%. Such high pseudocapacitive contribution reveals that the fast kinetics of TPQB electrode. The kinetics of TPQB cathodes were also analyzed by in situ electrochemical impedance spectroscopy (EIS) measurement (Figure S15, Supporting Information).^[^ ^54^ ^]^ As the cycle goes, the charge transfer resistance (*R*_ct_) gradually increases to stabilize, indicative of stable electrode/electrolyte interface formation during the cycle.

In view of fast kinetics and high‐capacity expression, the long‐term cycle performance of the TPQB electrode at a relatively high current density of 5 C was explored after activating at 0.2 C for 10 cycles (Figure 3h). After cycling for 500 times, the capacity retention remains as high as 87.7%. Moreover, after 1000 cycles, high‐capacity retention of 76.8% is still achieved. Except for initial cycles, the CEs close to 100%. TPQB demonstrates superior electrochemical performance in LIBs, surpassing all reported PQ‐based organic small‐molecule cathode materials while also exhibiting competitive capabilities comparable to state‐of‐the‐art PQ‐derived polymer cathode materials (Table S1, Supporting Information). These results clearly demonstrate that electrochemical performance of PQ‐based cathode materials for LIBs is greatly improved through our reasonable molecular structure engineering.

### 2.4 Reaction Mechanism of TPQB//Li Batteries

**Figure 4:**
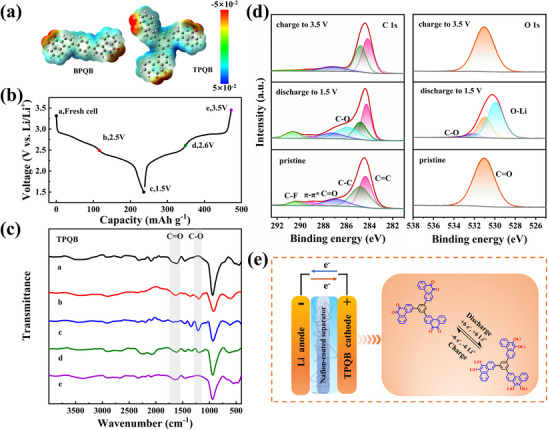
Mechanism study of TPQB cathode. (a) ESP images of BPQB and TPQB molecules. (b) GCD profiles of the TPQB cathode at 0.2 C with marked points and (c) their corresponding ex situ FTIR spectra. (d) Ex situ C 1s and O 1s XPS of TPQB cathodes at the pristine, fully discharged, and fully charged states. (e) Illustration of storage mechanism for TPQB in LIBs.

Generally, the carbonyl group serves as the redox reaction site of quinone‐based materials. To prove this in theory, electrostatic potential (ESP) calculations were performed. As shown in **Figure** **4**a, the C ═ O groups of BPQB and TPQB, located in the red regions with minimal ESP value, are identified as potential electrophilic centers for Li⁺ adsorption.^[^ ^34^ ^]^ To experimentally elucidate the charge storage mechanism, ex situ FTIR measurements were first conducted. TPQB electrodes in different charge and discharge states were retrieved (Figure 4b) and analyzed. As shown in Figure 4c, the peak at 1655 cm^−1^ is attributed to vibration absorption of C ═ O group, whose intensity gradually decreases during the discharge process and vanishes entirely at the fully discharged state (1.5 V), confirming the participation of all carbonyl groups. Upon charging, the peak of C ═ O group is recovered progressively, demonstrating good reversibility. In contrast, the peak at 1100 cm^−1^ ascribed to C─O bond shows the inverse trend. These results reveal that the carbonyl group is a reversible active site of TPQB cathode in LIBs.

To further verify the reversible conversion of C ═ O group, X‐ray photoelectron spectroscopy (XPS) of TPQB electrodes with different charging and discharging states in C 1s and O 1s regions were tested (Figure 4d).^[^ ^46^ ^]^ The high‐resolution C 1s spectra of TPQB cathode were analyzed first. The primitive TPQB cathode exhibits five distinct peaks at 284.1, 284.8, 287.2, 288.6, and 290.4 eV, which are respectively assigned to C ═ C, C─C, C ═ O, *π*−π*, and C−F. Among them, the existence of *π*−π* peak corresponds to the intermolecular π‐π stacking interactions, further verifying the low stereoscopicity of TPQB. The utilization of PVDF as binder introduces the C−F peak.^[^ ^55^, ^56^ ^]^ During the discharge process, a new peak of C─O appears ≈285.9 eV in the fully discharged state (1.5 V). Upon recharging to 3.5 V, the intensity of the C ═ O peak is restored, while the C─O peak disappears. Similarly, the change of O 1s peaks can also prove the reversible conversion of C ═ O group. As shown in Figure 4d, the fully discharged TPQB electrode appears new peaks of C─O and O─Li at 534.8 and 531.6 eV, respectively. After the charging process, the peaks of C─O and O─Li vanish, while that of C ═ O appears. These changes collectively demonstrate the reversible lithiation/delithiation process of C ═ O group during cycling. Therefore, the reaction mechanism of TPQB//Li battery is drawn in Figure 4e. During discharge, electron transfer occurs between TPQB cathode and the lithium metal anode through the external circuit, triggering the reduction of C ═ O groups to the corresponding enolate form (C─O⁻). It is accompanied by the sequential insertion of six lithium ions into TPQB, ultimately leading to the formation of Li_6_TPQB. Conversely, during charge, the reverse reaction occurs. Additionally, DFT calculations were also performed to analyze the reactivity order of the six carbonyl groups in TPQB toward lithium ions (Table S2 and Figure S16, see Supporting Information for details).

### 2.5 Electrochemical Performance of TPQB‐based Zn‐organic Batteries

Aqueous rechargeable Zn‐organic batteries have emerged as a promising energy storage technology, owing to their inherent advantages including low cost, operational safety, and environmental friendliness.^[^ ^57^, ^58^, ^59^ ^]^ Despite PQ‐based molecules are featured with higher voltage and superior zinc‐ion coordination affinity, their electrochemical performance remains to be enhanced, primarily due to their susceptibility to dissolution in aqueous electrolytes.^[^ ^26^, ^28^ ^]^ In view of TPQB displaying outstanding electrochemical performance for LIBs, it is proposed that TPQB would also be a promising organic cathode material for AZIBs. Herein, the electrochemical performance of the TPQB cathode was also evaluated in coin‐type cells with Zn metal as anode, and 3 m zinc trifluoromethanesulfonate salt (Zn(OTf)_2_) in deionized H_2_O served as electrolyte. All electrochemical tests are performed at 0.25–1.6 V (vs Zn/Zn^2+^).

**Figure 5:**
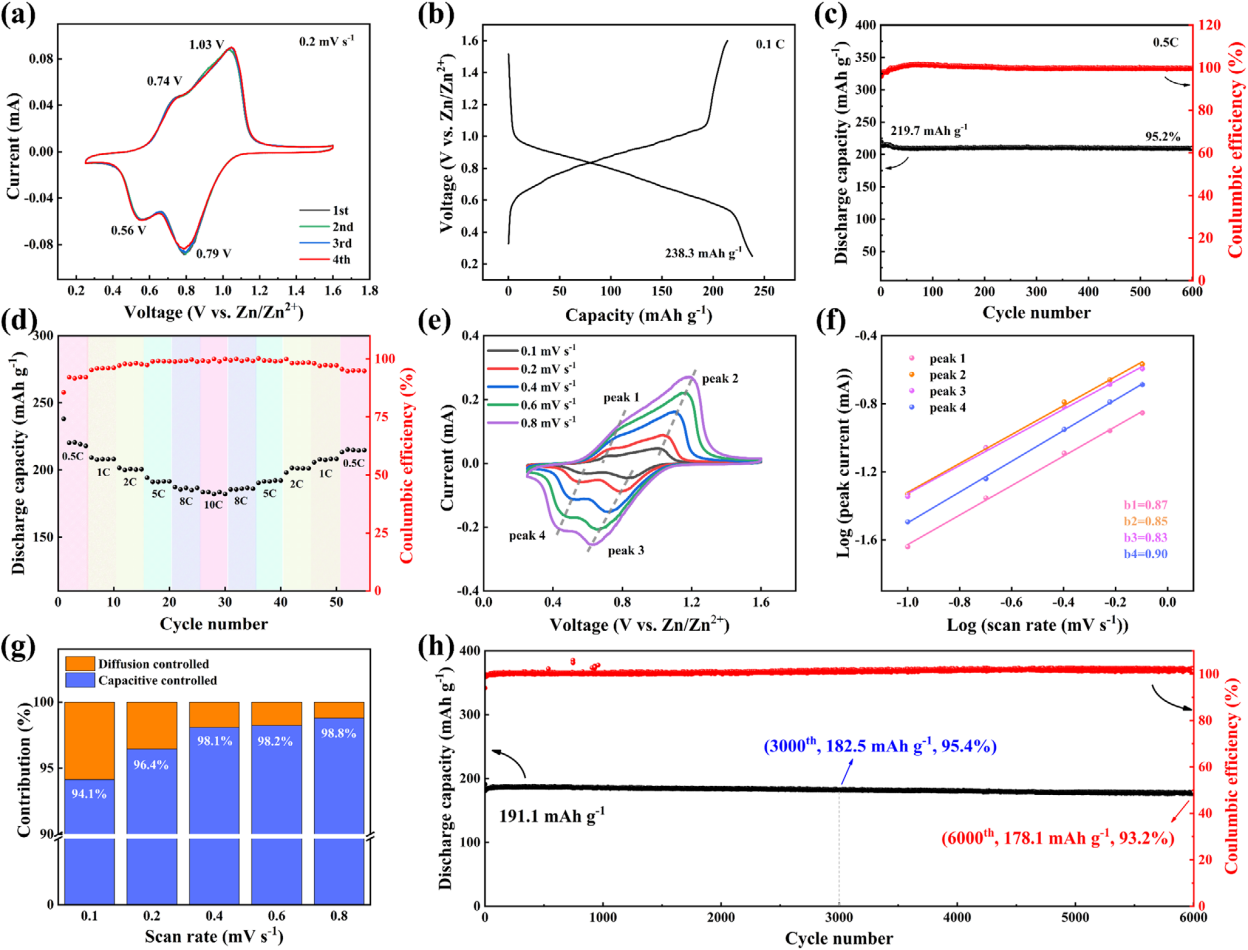
Electrochemical performance of TPQB cathodes in AZIBs. (a) CV curves at a scan rate of 0.2 mV s^−1^. (b) GCD profiles of first cycles at 0.1 C. (c) Cycle performance at 0.5 C. (d) Rate performance at different current densities. (e) CV curves at different scan rates and (f) the corresponding plots of log(*i*, peak current) versus log(*v*, scan rate) for CV curves at different scan rates. (g) Capacity contribution ratios of diffusion control (blue) and surface control (orange) at different scan rates, and (h) long‐term cycle performance at a high current density of 5 C.

The electrochemical property of TPQB electrode was initially studied by CV test. As depicted in **Figure** **5**a, the CV profiles exhibit two distinct pairs of redox peaks at 0.56 V/0.74 V and 0.79 V/1.03 V (vs Zn/Zn^2+^), corresponding to the two‐stage redox transitions of TPQB. Notably, the profiles are highly overlapping in the initial four cycles, confirming their high stability and electrochemical reversibility within the aqueous electrolyte system. GCD curve at 0.1 C (Figure 5b) delivers an initial specific capacity of 238.3 mAh g⁻^1^, with contribution from Ketjen black determined as 12.5 mAh g⁻^1^ (Figure S17, Supporting Information). The average operating voltage is ≈0.80 V (vs Zn/Zn^2+^), which significantly surpasses that of para‐quinone analogs (e.g., AQ, ≈ 0.50 V).^[^ ^28^ ^]^ As shown in Figure 5c, TPQB cathode exhibits outstanding cycling stability at 0.5 C, with 95.2% capacity retention maintained over 600 cycles, preliminarily establishing TPQB as a superior organic cathode material for AZIBs. The observed CEs in the initial cycles are comparatively low, which can be attributed to prolonged exposure to high voltage during cycling at low current densities, with probable side reactions such as electrolyte decomposition.

The rate capability of TPQB cathode for AZIBs was also evaluated, as illustrated in Figure 5d, it displays specific capacities of 220.6, 208.1, 200.7, 191.2, 186.7, and 182.2 mAh g^−1^ at progressively increased current densities of 0.5, 1, 2, 5, 8, and 10 C, respectively. Notably, the high‐capacity retention at 10 C reaches 82.6% relative to its initial capacity at 0.5 C. When the current density is gradually restored to 0.5 C after high‐rate cycling, the capacity ultimately recovers to 215.2 mAh g^−1^ (97.5% of the initial value), further proving the outstanding redox reversibility and stability of TPQB electrode in the aqueous system. The well‐maintained voltage plateaus observed in GCD profiles at various current densities imply the stable charge transfer kinetics and also superior rate performance of the TPQB electrode (Figure S18, Supporting Information).

To explore the reaction kinetics of TPQB cathode in AZIBs, CV measurement was conducted at various scan rates from 0.1 to 0.8 mV s^−1^. As shown in Figure 5e, the CV profiles maintain their characteristic curve shape without obvious peak distortion at elevated scan rates, further corroborating the superior rate performance and fast reaction kinetics of TPQB. Furthermore, the relationship between peak current (*i*) and voltage scan rate (*v*) was also performed using the formula *i* = *a v^b^*,^[^ ^60^ ^]^ where the calculated *b* values for the four redox peaks are 0.87, 0.85, 0.83, and 0.90, respectively (Figure 5f), indicating fast pseudocapacitive‐dominated electrochemical behavior. Capacity contribution ratios of diffusion and surface control at different scan rates were also calculated (Figure 5g; Figure S19, Supporting Information).^[^ ^61^ ^]^ At a low scan rate of 0.1 mV s^−1^, the ratio of capacitive control is as high as 94.1%, and it gradually rises to 98.8% as the scan rate increases to 0.8 mV s^−1^, highlighting its high pseudocapacitive contribution. The fast kinetics of TPQB cathode for AZIBs was also reflected by the GITT and EIS measurements (Figures S20 and S21, Supporting Information).^[^ ^62^, ^63^ ^]^

Long‐term cycle performance of TPQB electrode for AZIBs was investigated at a high current density of 5 C. As shown in Figure 5h, after 3000 cycles, TPQB electrode sachieves very high capacity retention up to 95.4%. Remarkably, even after ultra‐long charge‐discharge cycles of 6000 times, the capacity retention still maintains 93.2% of the initial capacity, exhibiting superb cycle performance. It achieves a minor average capacity fading rate of only 0.001% per cycle. The CEs are almost 100% during cycling. This performance surpasses all reported PQ‐based cathodes in AZIBs (Table S3, Supporting Information), which stems from our rational molecular engineering that strategically reduces molecular solubility and ensures the stability of electrode structure during long‐term cycles. The exceptional cycling stability demonstrated primarily stems from aqueous electrolyte, which offers some advantages over conventional organic electrolytes used in LIBs. First, the intrinsically low dissolution tendency of organic species in aqueous media fundamentally improves the stability of electrode structure, leading to superior cycle performance.^[^ ^35^ ^]^ Second, the OTf⁻ anion can maintain zinc anode reactivity and structural integrity by modulating the hydrogen‐bond network and suppressing zinc anode passivation.^[^ ^64^ ^]^ Thirdly, the smaller ionic radius of Zn^2^⁺ (0.74 Å) compared to Li⁺ (0.76 Å) facilitates the preservation of structural integrity during cycling.^[^ ^14^ ^]^

### 2.6 Reaction Mechanism of TPQB//Zn Batteries

**Figure 6:**
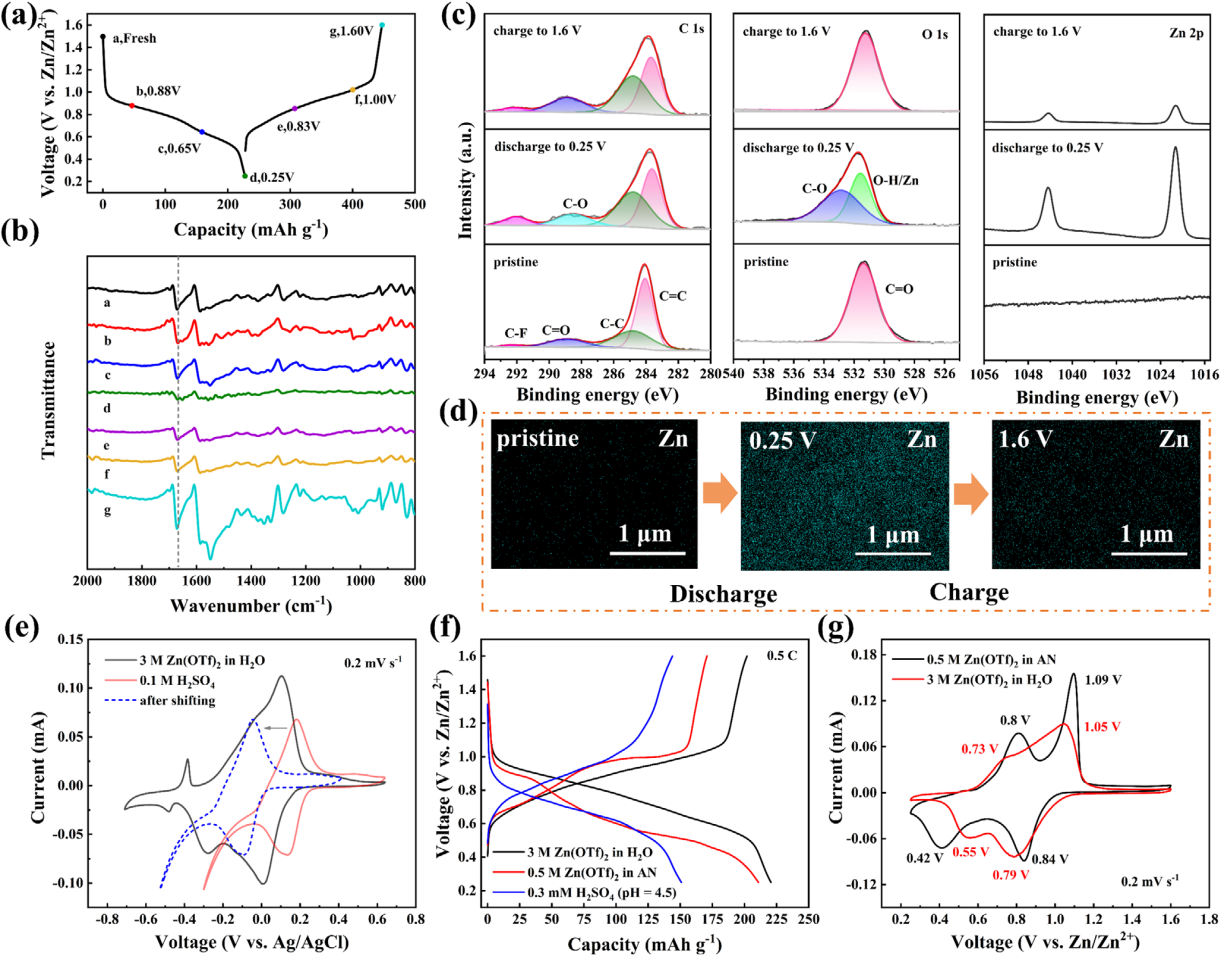
Mechanism study of TPQB cathodes in AZIBs. (a) GCD profiles of the TPQB cathode at 0.2 C with marked points and their corresponding ex situ FTIR spectra (b). (c) Ex situ C 1s, O 1s, and Zn 2p XPS spectra and (d) EDS mapping of Zn elemental distributions of TPQB cathodes at the pristine, fully discharged (0.25 V), and fully charged (1.6 V) states. (e) CV curves of TPQB measured by typical three‐electrode systems at 0.2 mV s^−1^. (f) GCD and (g) CV curves of TPQB in different electrolytes.

To Figure out the charge storage mechanism of TPQB electrode in AZIBs, the FTIR, XPS, and SEM measurements were performed. Electrodes were systematically harvested at different charge/discharge states (**Figure** **6**a) and analyzed by ex situ FTIR technique. As shown in Figure 6b, the characteristic C ═ O stretching vibration peak at 1672 cm⁻¹ exhibits progressive attenuation during discharge, followed by restoration upon charging. XPS spectra of TPQB electrodes at various electrochemical states (pristine, discharged to 0.25 V, and charged to 1.6 V) reveal dynamic chemical evolution in C 1s, O 1s, and Zn 2p regions (Figure 6c).^[^ ^65^ ^]^ In the C 1s region, the pristine TPQB cathode exhibits four distinct peaks at 283.9 eV (C ═ C), 284.8 eV (C─C), 288.9 eV (C ═ O), and 292.3 eV (C─F), corresponding to molecular structure and electrode composition. After discharge to 0.25 V, the C ═ O peak disappears with the concurrent emergence of C─O peak (288.5 eV). When charged to 1.6 V, C ═ O peak is recovered. Moreover, the O 1s spectra show reversible transformation between C ═ O groups and C─O/O─X (anti‐cation, e.g. O─Zn/O─H) species (532.9 and 531.6 eV, respectively) during cycling. These results indicate that carbonyl groups of TPQB are the active sites. Additionally, the Zn 2p XPS spectra of TPQB electrode at 0.25 V verify the intercalation of Zn cations, while significantly reducing after being recharged to 1.6 V. The EDS mapping images were performed to further prove the involvement of zinc ions during the electrochemical process (Figure 6d). At fully discharged state, Zn elements appear uniformly in large quantities, while it almost disappear at the fully charged state. These results support the remarkably reversible zinc‐ion coordination/de‐coordination behavior in the TPQB//Zn battery system.

Generally, co‐participation of hydrogen ions and zinc ions during redox reactions often occurs in quinone cathode materials for AZIBs.^[^ ^66^, ^67^ ^]^ To demonstrate the involvement of proton storage, systematic electrochemical analyses of TPQB electrodes in different electrolytes were conducted by CV and GCD measurements. As shown in Figure 6e, CV curves of TPQB in 0.1 m H_2_SO_4_ electrolyte and 3 m Zn(OTf)_2_ electrolyte were measured by a typical three‐electrode system using Pt metal as counter electrode and Ag/AgCl (soaked in saturated KCl solution) as reference electrode (0.197 V vs SHE).^[^ ^68^ ^]^ In 3 m Zn(OTf)_2_ electrolytes, three pairs of redox peaks are observed. In 0.1 m H_2_SO_4_ electrolyte, only one pair of redox peaks appears at 0.128 and 0.186 V (vs Ag/AgCl), which is related to the protonation/deprotonation of C ═ O group. The theoretical CV curves in dilute H_2_SO_4_ solution (pH = 4.5) calculated via the Nernst equation (See Supporting information for details) reveal potential‐shifted peaks at −0.092 and −0.050 V (vs Ag/AgCl), showing partial overlap with CV curves measured in 3 m Zn(OTf)_2_ electrolyte. This result confirms the co‐participation of H^+^ and Zn^2+^ during the charge storage process.

To further elucidate the co‐insertion behavior of H^+^ and Zn^2+^, comparative electrochemical evaluations were tested in three distinct electrolytes: 3 m Zn(OTf)_2_/H_2_O (contains both H^+^ and Zn^2+^), 0.5 m Zn(OTf)_2_/AN (only Zn^2+^), and 0.3 mm H_2_SO_4_ (only H^+^). As shown in Figure 6f, the TPQB cathode delivers a capacity of 210.9 mAh g^−1^ in 0.5 m Zn(OTf)_2_/AN, which is very close to the capacity of 220.6 mAh g^−1^ in 3 m Zn(OTf)_2_/H_2_O electrolyte. In contrast, the capacity in 0.3 mm H_2_SO_4_ is only 150.6 mAh g^−1^. These results suggest that zinc ions play a dominant role during the charge storage process in 3 m Zn(OTf)_2_. Besides, it is further proved by CV curves in 3 m Zn(OTf)_2_ and 0.5 m Zn(OTf)_2_/AN electrolyte (Figure 6g), where redox peaks show a good overlap. Overall, the electrochemical reaction process of TPQB electrode for AZIBs is the co‐participation of H^+^ and Zn^2+^, wherein Zn^2^⁺ insertion/extraction processes dominate.

## 3 Conclusion

To improve the electrochemical performance of PQ derivatives in organic batteries, we synthesized two PQ‐based organic compounds, namely BPQB and TPQB, through molecular engineering to suppress dissolution. Electrochemical tests manifest that TPQB is a superior cathode material, demonstrating remarkable cycling stability and rate performance for both LIBs and AZIBs. In LIBs, the TPQB cathode achieves 76.8% capacity retention after 1000 cycles at 5 C, coupled with exceptional rate capability delivering 186.7 mAh g⁻¹ at an ultrahigh current density of 40 C. More remarkably, when implemented in AZIBs, the TPQB cathode delivers an ultra‐long cycle life, with a capacity retention rate of 93.2% after 6000 cycles at 5 C. These investigations provide valuable insights into the rational design and application of PQ‐derived organic molecules in diverse organic batteryx systems.

## Conflict of Interest

The authors declare no conflict of interest.

## Supporting information



Supporting Information

## Data Availability

Research data are not shared.
