# Supplementary material for: Molecular Engineering Empowers Phenanthraquinone Organic Cathodes with Exceptional Cycling Stability for Lithium‐ and Aqueous Zinc‐Ion Batteries
Source: Adv Sci (Weinh). 2025 Sep 11;12(39):e06749. doi: 10.1002/advs.202506749 (PMC12533210; doi:10.1002/advs.202506749)
Supplement: Supplementary file 1 — Supporting Information [file ADVS-12-e06749-s001.docx]

Supporting Information

Molecular Engineering Empowers Phenanthraquinone Organic Cathodes with Exceptional Cycling Stability for Lithium- and Aqueous Zinc-Ion Batteries

Susu Li, Haoyu Zhang, Jixing Yang,* Yunhua Xu, and Yuesheng Li

**Experimental Section**

**Materials**

All raw materials and reagents are purchased commercially without further processing. 3-bromo-9,10-phenanthrenedione (96%), camphorsulfonic acid (99%), ethylene glycol (AR, ≥99.5%), 9,10-phenanthrenequinone (98%), 1,4-dioxane (99.7%, extra dry), 1,3,5-benzenetriboronic acid tris(pinacol) ester (98%), 1,4-benzenediboronic acid bis(pinacol) ester (97%), potassium carbonate (99%), tetrakis(triphenylphosphine)palladium (99%), all from Anhui Zesheng Technology Co., Ltd. Toluene, methanol, petroleum ether and ethyl acetate (AR, Tianjin Yuanli Chemical Co., Lt dj), ultrapure water (Beyotime), trifluoroacetic acid (99%, Anhui Zesheng Technology Co., Ltd).

**Synthesis of** **3-bromophenanthrene-9,10-di(ethyleneglycol)ketal (BPQ-EGK)**

Camphorsulfonic acid (CAS) (0.31 g, 1.33 mol) and 3-bromo-9,10-phenanthrenedione (BPQ) (1.30 g, 4.55 mmol) were transferred to a dry 350 mL Schlenk tube under the nitrogen atmosphere. Ethylene glycol (EG) (35 mL) and extra dry 1,4-dioxane (70 mL) were subsequently added to the reaction mixture. The solution was then stirred at 140 ℃ for 12 h. After cooling to room temperature, the mixed solution was poured into deionized water. The precipitate was collected by filtration and washed with H_2_O (3×100 mL) and dried overnight at 80 ℃ to afford the earthy yellow powder of 1.41 g. Then the product was further purified by column chromatography (petroleum ether/ethyl acetate = 20:1, V/V), obtaining the white powder (BPQ-EGK) in the yield of 1.0 g (77.0%).

**Synthesis of TPQB**

**(1) Step 1:** Suzuki coupling reaction. Under the protection of nitrogen atmosphere, molecule BPQ-EGK (0.7 g, 1.87 mmol), 1,3,5-benzenetriboronic acid tris(pinacol) ester (TTDB) (0.21 g, 0.46 mmol), K_2_CO_3_ (0.193 g, 1.4 mmol) and Pd^0^(PPh_3_)_4_ (54 mg, 0.04 mmol) were added in a fully dried Schlenk flask. Subsequently, 40 mL toluene, 4 mL methanol, and 1 mL deionized water were added into the flask and the mixture was stirred at 100 ℃ under nitrogen for 12 h. while still hot, the mixture was filtered to collect the precipitation. Then the precipitation was successively washed with toluene, deionized water, and ethanol, successively, followed by drying in the vacuum oven at 80 ℃ overnight. Finally, the product was purified by Soxhlet extraction to obtain the white powder, TPQB-EGK, in the yield of 0.338 g (75.2%). The synthesis route is referred to the literature: *ChemSusChem* **2020**, *13*, 2436.

**(2) Step 2:** Deprotection reaction. TPQB-EGK (0.3 g) was added to a 100 mL flask containing a mixture of trifluoroacetic acid (TFA) and water (9:1, V/V, 50 mL). The mixture was stirred at 100 ℃ for 2 days. After cooling to room temperature, the mixture was poured into 1000 ml of ice water. The resulting precipitate was collected by filtration and dried to obtain yellow solid in the yield of 0.218 g (88.6%).

**Synthesis of BPQB**

The synthesis of BPQB is similar to TPQB, except that the raw materials are different. The yield of BPQB-EGK and BPQB are 0.364 g (85.4%) and 0.197 g (89.5%), respectively.

**Preparation of** **Electrodes and Fabrication of LIBs**

The PQ/BPQB/TPQB electrodes for LIBs were prepared by mixing active materials, Graphene (GR) and polyvinylidenefluoride (PVDF) in the ratio of 6:3:1 (wt%). The active materials and GR (6:3) were ground in a mortar for 45 minutes firstly, then 10 wt% PVDF was added and ground for 20 minutes, and the moderate N-methylpyrrolidone (NMP) was added to form a well-dispersed slurry. The mixture was stirred for 20 minutes and then casted onto a carbon-coating aluminum foil by a medical knife. After drying at 60 °C for 6 hours, the electrode was punched into circular discs with a diameter of 9 mm. The Nafion-coated polypropylene (PP) (Celgard 2500, LLC Corp., USA) is employed as the separators (Its preparation is referred to the our previous report: *Adv. Mater.* **2022**, *34*, 2107226). The 2 M lithium bis(trifluoromethanesulfonyl)imide (LiTFSI) and 0.6 M poly(ethylene oxide) (PEO) in 1,3-dioxolane(DOL)/1,2-dimethoxyethane(DME) (V/V = 1/1) is served as the electrolyte. Using the lithium metal as anode material and the BPQB/TPQB electrode as cathode material, the coin-type (CR2032) cells are assembled in an argon-filled glove box (O_2_ < 0.1 ppm, H_2_O < 0.1 ppm).

**Preparation of Electrodes and Fabrication of AZIBs**

The TPQB electrodes for AZIBs were prepared by mixing TPQB, conductive carbon (Ketjen black, KB) and PVDF in the ratio of 6:3:1 (wt%). The TPQB and KB (6:3) were ground in a mortar for 45 minutes firstly, then 10 wt% PVDF was added and ground for 20 minutes, and then the moderate NMP was added to form a well-dispersed slurry. The mixture was stirred for 20 minutes and then casted onto a titanium foil by a medical knife. After drying at 60 °C for 6 hours, the electrode was punched into circular discs with a diameter of 9 mm. The Whatman glass fiber (Whatman GF/D) was used as the separators.

The 3 M zinc trifluoromethanesulfonate salt (Zn(OTf)_2_) in DI H_2_O was served as the electrolyte. The coin-type (CR2032) cells are assembled by using the Zn foil (50 μm) as anode material and the TPQB electrode as cathode materail. The typical three-electrode systems are assembled by Pt metal as counter electrode and Ag/AgCl (soaked in saturated KCl solution) as reference electrode (0.197 V vs. SHE), the TPQB electrode as working electrode.

**Material Characterizations**

The ^1^H NMR spectra was collected on a Bruker AVANCE III HD 400 MHz with CDCl_3_ as the deuterated solvent to verify the structure of the moleculars. The bonding and group characteristics of BPQB and TPQB powers and electrodes were investigated by FTIR (BRUKER ALPHA). The MS spectra was collected on a MicrOTOF-Q II to determine the molecular mass. The TG curves of the BPQB and TPQB materials are recorded from TG209F3 thermogravimetric analyzer at a heating rate of 10 °C min^-1^ in N_2_ atmosphere. The solid-state ^13^C NMR spectras of BPQB and TPQB were recorded from JEOL JNM ECZ600R. The X-ray diffraction (XRD) patterns were collected on Rigaku Ultima IV with Cu k α radiation (λ = 1.5406 Ȧ, 40 KV and 40 mA). The microstructures and chemical composition of the materials and electrodes were observed by Scanning Electron microscope (SEM, S-4800, Japan) and Energy Dispersive Spectrometer (EDS, HORIBA EX350). The ultraviolet-visible spectroscopy (UV-vis, Shimadzu UV-3600 plus, Japan) was used to explore the solubility of the materials. The chemical composition of the electrodes was analyzed using X-ray photoelectron spectroscopy (XPS, K-Alpha+).

**Electrochemical Measurements**

Cyclic voltammetry (CV) measurement and electrochemical impedance spectroscopy (EIS) at 10^5^-0.01 Hz frequencies with an amplitude of 5 mV were performed on a Solartron 1470E electrochemical workstation (Solartron Metrology, UK). The constant current charge-discharge curves, cycle performance and rate performance were tested on the LAND battery test system (CT2001A, China) and the NEWARE battery testing system. In the GITT measurements, a series of 0.1 C charge/discharge current pulses with a duration of 30 min were applied, followed by an open circuit time of 1 h for each pulse. The CV of typical three-electrode systems was tested at electrochemical workstation (CHI-600E).

**Theoretical calculation**

All calculations based on density functional theory (DFT) were carried out using the Gaussian 06 software. The optimized geometry and frequency of the structures were calculated at the B3LYP/6-31g level. Contents relating to molecular electrostatic potential (ESP) were analyzed by using Gaussian 06.

**Calculation based on Nernst Equation**

The calculation process refers to the literature: *Adv. Mater.* **2020**, *32*, e2000338.

Electrode reaction:

$$TPQB+6H^{+}\rightleftharpoons H_{6}TPQB$$

Nernst Equation:

$$\varphi=\varphi^{\theta}+\frac{2.303RT}{nF}\log_{10} \frac{\left[ TPQB \right]{[H^{+}]}^{6}}{[H_{6}TPQB]}$$

Where *φ* is electrode potential; *φ*^θ^ is the standard potential; *R* is the universe gas constant: 8.314 J K^-1^ mol^-1^; *T* is the temperature: 299.15 K; *n* is the electron transfer numbers; *F* is the Faraday constant: 96500 C mol^-1^. The activity of solid TPQB and H_6_TPQB is considered as 1, and *n* is 6, the equation can be further simplified as:

$$\varphi=\varphi^{\theta}+0.0594\log_{10} [H^{+}]$$

Thus, the potential difference of TPQB in a 0.1 M H_2_SO_4_ solution and in a pH = 4.5 aqueous solution can be calculated as:

$$\Delta\varphi=0.0594\log_{10} \frac{{[H^{+}]}_{1}}{{[H^{+}]}_{2}}=0.0594\log_{10} \frac{0.2}{{10}^{-4.5}} V=0.226 V$$

**Figures and Tables**

**Figure S1.** ^1^ H NMR spectra of the BPQ-EGK.

The peaks at chemical shifts (δ) = 8.03, 7.85, 7.77, 7.61, 7.49 ppm were attributed to H_a_, H_b_, H_c_, H_d_, H_e_ atoms of phenanthrene ring, respectively. The double peaks at δ = 4.20, 3.67 ppm correspond to H_f_ atoms of cyclic ketal structure. The area ratio of above-mentioned six peaks is 1 : 1 : 1 : 1 : 3 : 8 (H_a_ : H_b_ : H_c_ : H_d_ : H_e_ : H_f_), consistent well with theoretical value.

**Figure S2.** ^1^H NMR spectra of the BPQB-EGK.

The single peak at chemical shift (δ) of 8.17 ppm is assigned to the H atoms on the benzene ring (H_g_). The multiplet peaks at δ = 8.03, 7.86, 7.80, 7.77, 7.69, 7.50 ppm were attributed to H_a_, H_b_, H_c_, H_d_, H_e_, H_f_ atoms of phenanthrene ring, respectively. The double peaks at δ = 4.25, 3.73 ppm correspond to H_h_ atoms of cyclic acetal structure. The area ratio of above-mentioned eight peaks is 1 : 1 : 1 :1 : 2 : 1 : 2 : 8 (H_a_ : H_b_ : H_c_ : H_d_ : H_e_ : H_f_ : H_g_ : H_h_), consistent well with theoretical value.


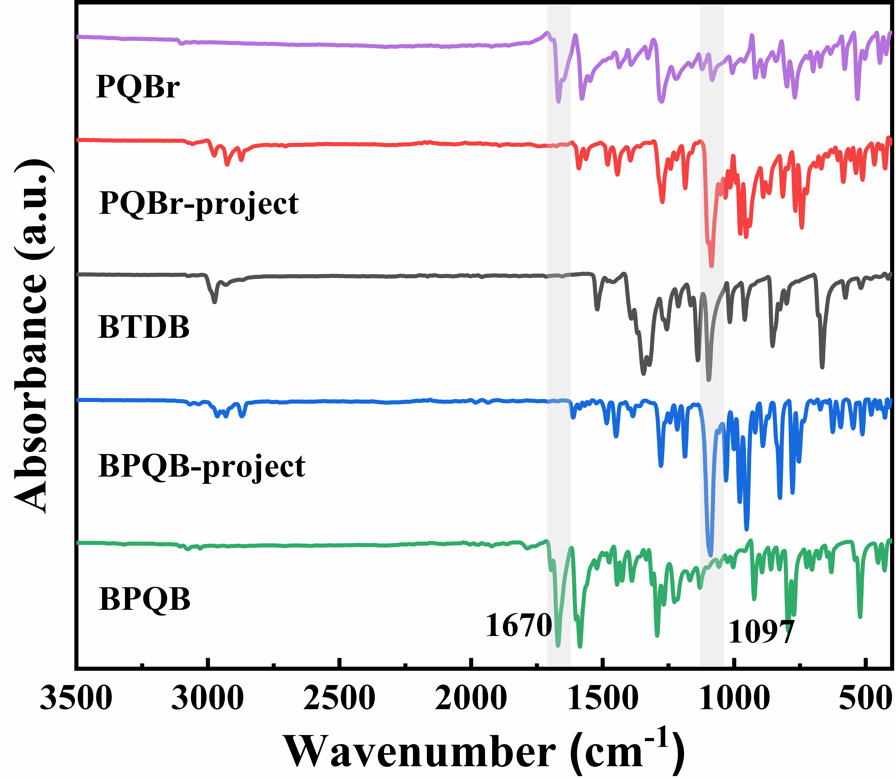


**Figure S3.** FTIR spectra of the reaction process of BPQB.


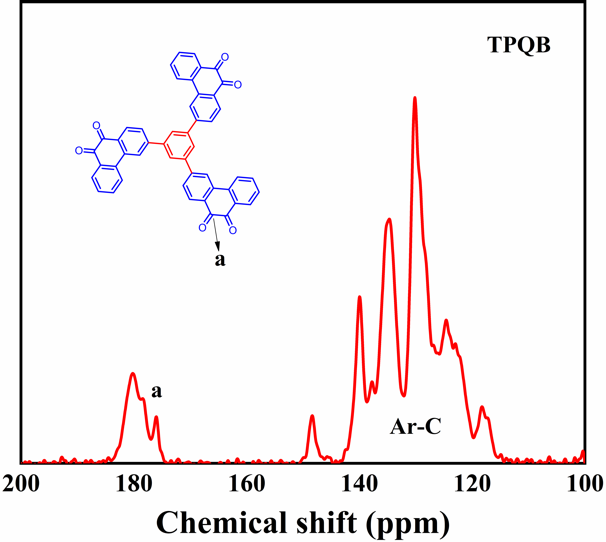

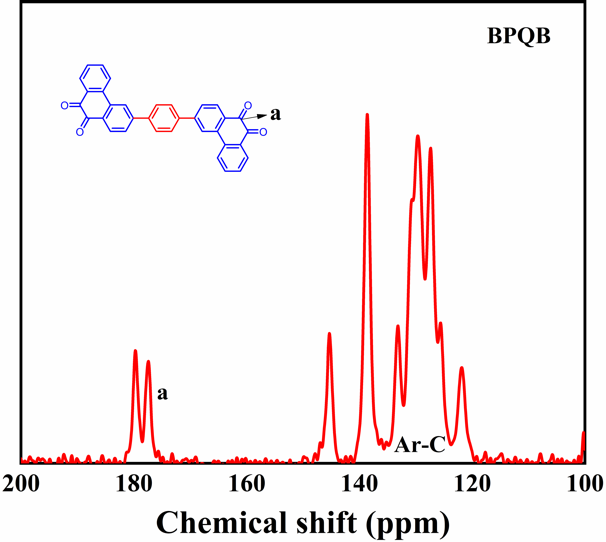


**Figure S4.** ^13^C solid-state NMR spectra of BPQB and TPQB.


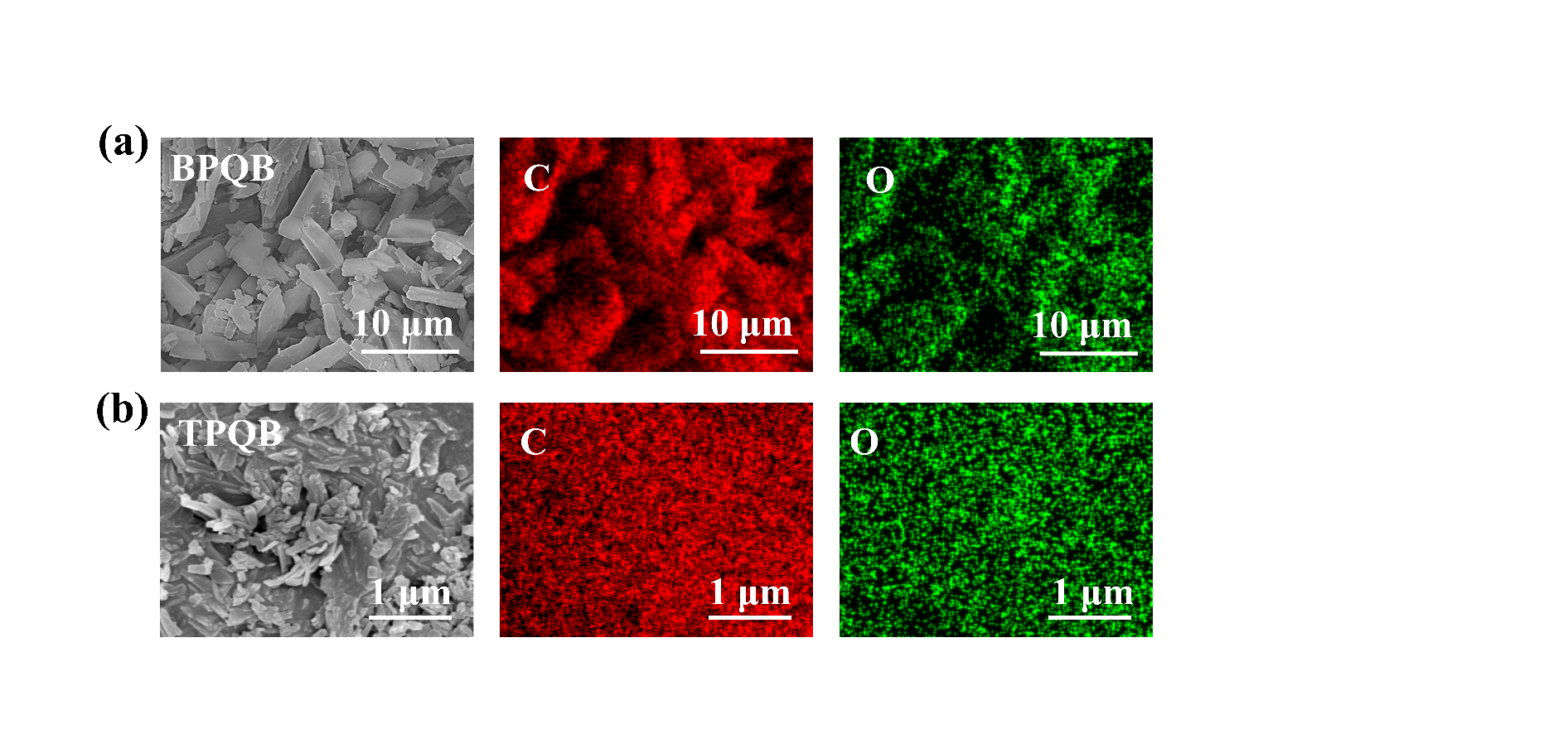


**Figure S5.** EDS mapping of C and O elements of the BPQB (a) and TPQB (b).

**Figure S6.** Optimized geometries of BAQB and TAQB by DFT calculations.


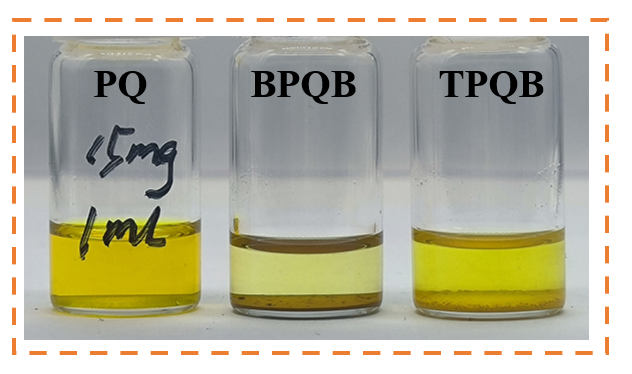


**Figure S7.** Photographs of 15 mg PQ, BPQB and TPQB powders in 1 mL DOL/DME (V:V = 1:1).


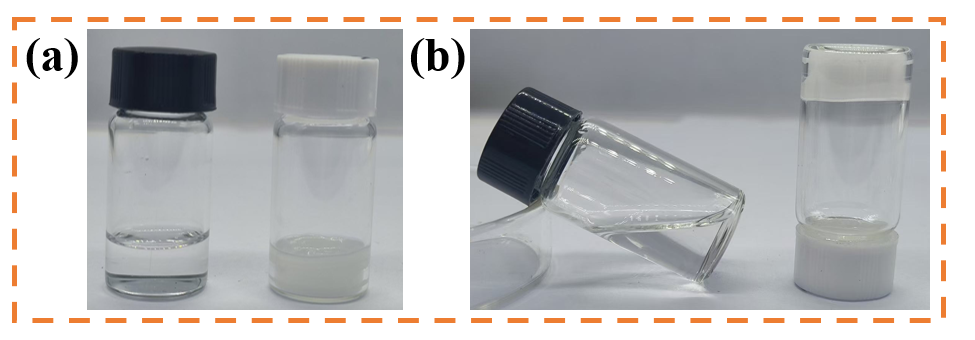


**Figure S8.** The visualization experiment of the formation of the gel polymer. (a) photo of liquid electrolyte (1 M LiTFSI in DOL/DME (V/V = 1:1)) and gel polymer electrolyte (with Nafion applied to the inside of glass bottle). (b) photo of the tipping state.


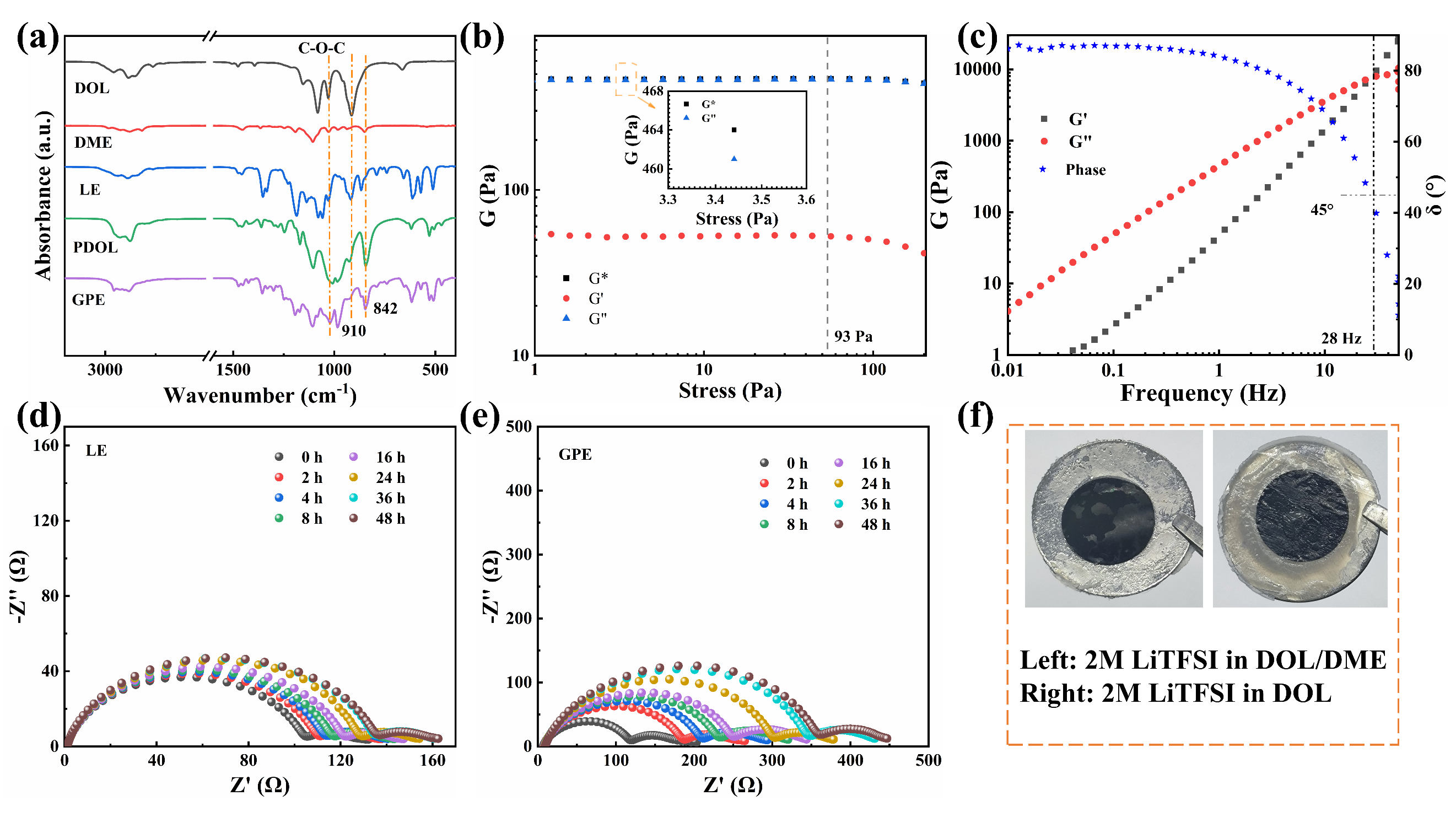


**Figure S9.** (a) FTIR of DOL, DME, LE, PDOL and GPE. (b-c) Rheological characterization of in-situ polymerized GPE. (d-e) EIS of LE and GPE electrolyte systems in Li-Li symmetric cells. (f) An illustration of gel formation in the cells.

The formation of GPE was investigated through FTIR spectroscopy (Figure S9a). To minimize interference from unpolymerized DME, the electrolyte (2 M LiTFSI in DOL) was subjected to Nafion-induced polymerization, yielding a polymer electrolyte system without DME (designated as PDOL). Comparative analysis with DOL reveals marked attenuation in the characteristic out-of-plane C-H vibrational mode intensity at 910 cm^-1^ for both PDOL and GPE systems. Concurrently, the emergence of a novel absorption band at 842 cm^-1^, corresponding to long-chain polymer vibrations, provides direct spectroscopic evidence of successful DOL polymerization.

To verify the solid‑like behavior of the formed polymer, the rheological characterization of in-situ polymerized GPE was performed (Figure S9b,c). To minimize interference from lithium salts and DME, the polymerization of pure DOL was initiated using Nafion. Measurements were conducted under the conditions: 60℃ with parallel plate (20 mm diameter, 1 mm gap spacing). Oscillation stress sweep analysis reveals a critical stress threshold of 93 Pa, below which the sample maintained linear viscoelastic behavior. Additionally, the material demonstrates minimal storage modulus (G') coupled with predominant viscous flow characteristics (G'' > G'), indicating fluid-like behavior under static conditions. Oscillation scanning tests within the linear viscoelastic regime (20 Pa) quantified viscoelastic transitions. Combined analysis of both experiments reveals the viscoelastic behavior of the polymeric material formed by Nafion-initiated DOL polymerization.

Subsequently, the impedance evolution of LE and GPE were tested in Li-Li symmetric cells. The LE system exhibited relatively minimal impedance variation over 48 hours. In contrast, the GPE system demonstrated a pronounced impedance increase, confirming successful gelation. To further verify the successful formation of GPE systems, 2M LiTFSI in DOL/DME (V/V = 1:1) and 2M LiTFSI in DOL electrolytes was used in cells. Both cells incorporated PP separators coated with Nafion. Following 24 hours of resting, obvious gel polymer are formed in both disassembled batteries, with particularly distinct polymer layer observed in the pure DOL-based system (Figure S9f).

**Figure S10.** GCD curves of graphene in 20 cycles.

The BPQB/TPQB electrodes were prepared by mixing active materials, graphene and PVDF in the ratio of 6:3:1 (wt%). The capacities were based on the active materials and the capacity contribution of graphene in the electrodes was subtracted. Figure S8 shows that capacity contributions of graphene. After cycling stabilization, the capacity is about 35 mAh g^-1^. The actual capacity of active materials was calculated using the following equation:

$$C_{actuality}=C_{measure}-\frac{30\%}{60\%}\times C_{conductive carbon}$$

Where, $C_{actuality}$ is the actual capacity of active materials, $C_{measure}$ is the measured capacity, $C_{conductive carbon}$ is the capacity of graphene (unit: mAh g^-1^).


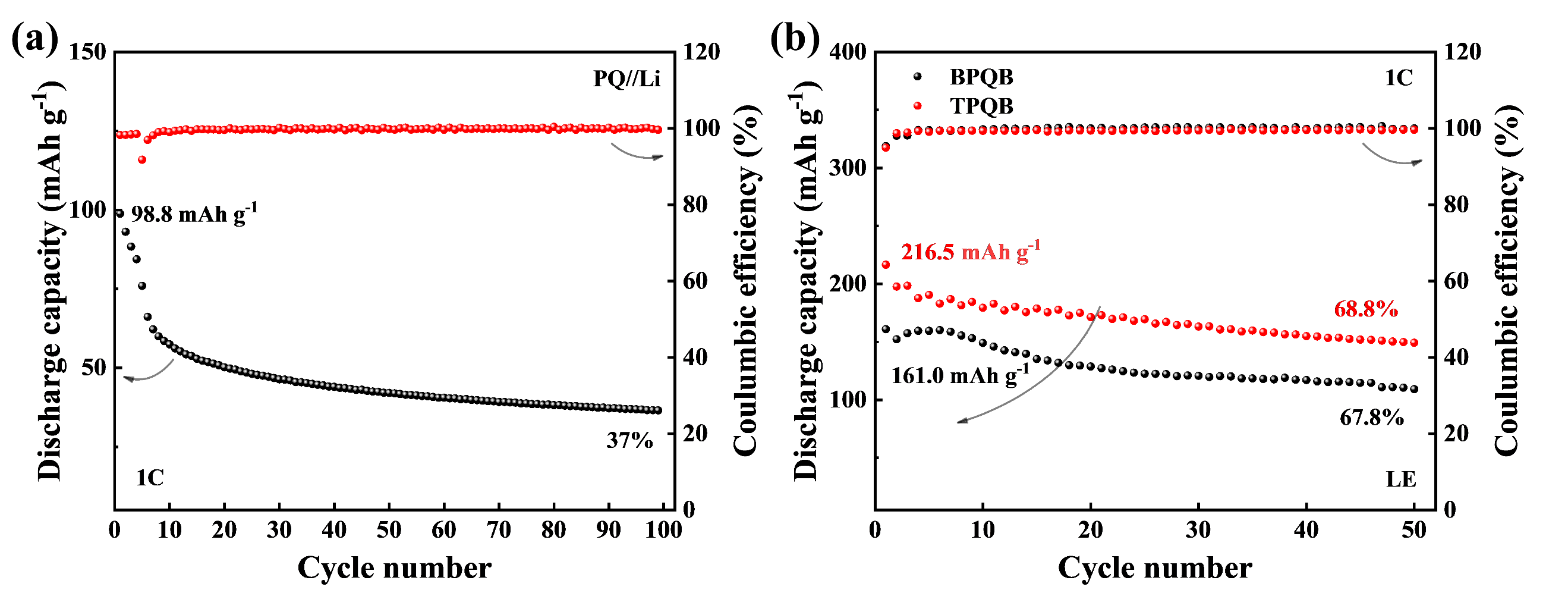


**Figure S11.** Cycle performance for LIBs. (a) PQ electrodes in gel electrolyte/functional separator system at 1 C. (b) BPQB and TPQB electrodes in common liquid electrolyte at 1 C.

The cycle performance of PQ cathode for LIBs was tested under the same gel electrolyte/functional separator system. As shown in Figure S11a, even with the gel polymer/functional separator system, the electrochemical performance of PQ electrodes remains very poor. Due to the dissolution of PQ molecules, the initial specific capacity of the PQ cathode is only 98.8 mAh g^-1^, which is much lower than its theoretical specific capacity of 257.7 mAh g^-1^. Meanwhile, after cycling for 100 times at 1 C, PQ cathode retains only 37% of its initial capacity. This proves the effectiveness of molecular design.

The electrochemical performance of BPQB and TPQB electrodes were testes in liquid electrolyte (LE) systems (Figure S11b). Using a 2 M LiTFSI in DOL/DME (V/V = 1:1) electrolyte, BPQB and TPQB electrodes exhibit reversible discharge capacities of 161.0 mAh g⁻¹ and 216.5 mAh g⁻¹ at 1C, respectively. Subsequent cycling stability tests reveal capacity retention rates of 67.8% and 68.8% after 100 charge-discharge cycles. The cycle performance in LE is inferior to that observed in gel electrolyte/functional separator system, thereby demonstrating the superior electrochemical stabilization effects of gel electrolyte/functional separator system.





**Figure S12.** GCD curves of TPQB cathode at different current densities.





**Figure S13.** GITT profiles and the diffusion coefficients of TPQB cathode.

The Li^+^ diffusion coefficients (*D*) was calculated using the following equation:

$$D=\frac{4}{\pi\tau}\left( \frac{m_{B}V_{M}}{M_{B}S} \right)^{2}\left( \frac{\Delta E_{s}}{\Delta E_{t}} \right)^{2}$$

in which τ is relaxation time, m_B_ is the mass of the active substance (unit: g), M_B_ is the relative molecular mass (unit: g/mol), V_M_ is the molar volume of electrode material (unit: cm^3^ mol^-1^), S is the effective surface area of contact between electrode and electrolyte (unit: cm^2^), and *ΔE*_S_ and *ΔE*_t_ are obtained according to the GITT curve.


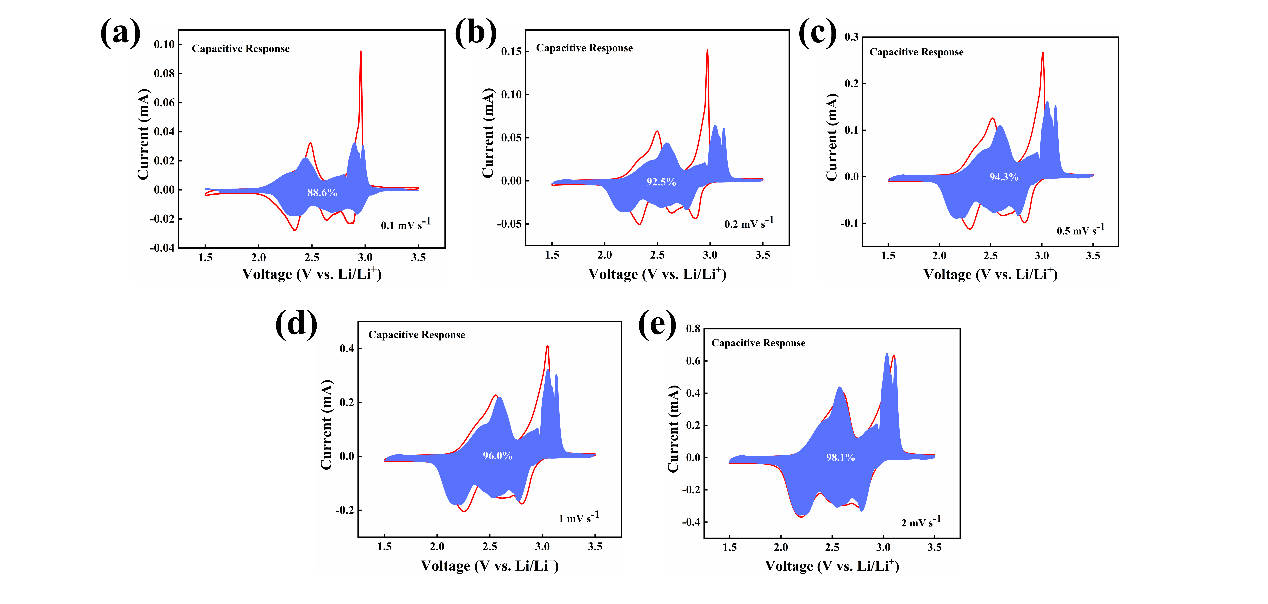


**Figure S14.** CV curves of TPQB cathode and corresponding capacitive contributions at scan rates of (a) 0.1, (b) 0.2, (c) 0.5 (d) 1, (e) 2 mV s^-1^.





**Figure S15.** Nyquist plots of TPQB cathode in different cycles.

**Table S1.** The performance comparison of TPQB//Li in this work with other organic electrode materials reported in the literatures.

| **Cathode Materials** | | **Theoretical**  **capacity**  **(mAh g^−1^)** | **Initial Discharge capacity (mAh g^-1^)** | **Cycle life, Capacity retention** | **Refs.** |
| --- | --- | --- | --- | --- | --- |
| **PQ-based small molecule electrode materials** | PhQ-CB  PhQ@SWCNTs | 258  258 | 148  Close to 258 | 50^th^, 10%  50^th^, 49%  at 100 mA g^-1^ | *Phys. Chem. Chem. Phys.* **2016**, *18*, 10411. |
|  | PQ/CMK-3 | 258 | 220 | 50^th^, 89%  at 0.1C | *Sci Rep.* **2014**, *10*, 7404. |
|  | PhQ/SWCNTs | 258 | 150 | 100^th^, 70%  at 100 mA g^-1^ | *ACS Omega* **2018**, *3*, 15598−15605. |
|  | PQ  LCPQ | 258  173 | 216  90 | 20^th^, 25%  20^th^, 105%  at 0.2 C | *J. Power Sources* **2014**, *260*, 211e217. |
|  | **TPQB** | **231** | **229.8** | **1000^th^, 76%**  **at 5C (1.15 A g^-1^)** | **This work** |
| **PQ-based polymer electrode materials** | DAPQ-COF50 | - | 162 | 3000^th^, 76%  at 2000 mA g^-1^ | *Adv. Energy Mater.* **2021**, *11*, 2101880. |
|  | PEPQ  PTPQ | 231  185 | 125  138 | 60^th^, 45%  100^th^, 62%  at 10 mA g^-1^ | *Int. J. Electrochem. Sci.* **2020**, *15*, 7774–7787. |
|  | PFQ  PFQ/rGO | 260  260 | 158  200 | 100^th^, 95%  500^th^, 91%  at 50 mA g^-1^ | *Chem. Mater.* **2018**, *30*, 5726−5732. |
|  | PQN | - | 110 | 100^th^, 100%  60C | *B. Chem. Soc. Jpn.* **2018**, *91*, 721-727. |

**Table S2.** Geometrical configurations (C, grey; H, white; O, red; Li, violet) and Gibbs free energies of TPQB molecule and its discharge products.

| **Molecule**  **name** | **Molecule**  **structure** | **Geometrical**  **configuration** | **Gibbs free**  **energy (Ha)** |
| --- | --- | --- | --- |
| TPQB |  | 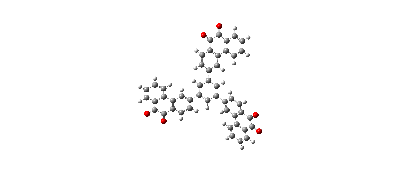 | -2293.855254 |
| LiTPQB |  | 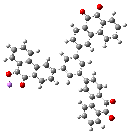 | -2301.489727 |
| Li_2_TPQB |  | 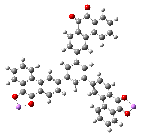 | -2309.093863 |
|  |  | 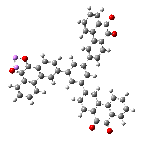 | -2309.070498 |
| Li_3_TPQB |  | 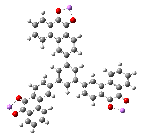 | -2316.754614 |
|  |  | 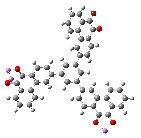 | -2316.703612 |
| Li_4_TPQB |  | 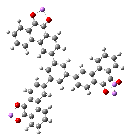 | -2324.306195 |
|  |  | 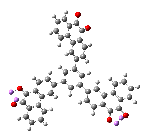 | -2324.283493 |
| Li_5_TPQB |  | 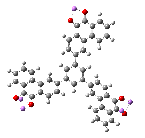 | -2331.915045 |
| Li_6_TPQB |  | 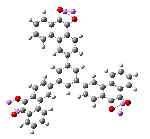 | -2339.494099 |

Our analysis, based on the above Gibbs free energy calculations, identified the intermediate molecule with the most stable structure (the lower Gibbs free energy) as the discharge product. The reaction sequence is shown in the Figure S16 below.


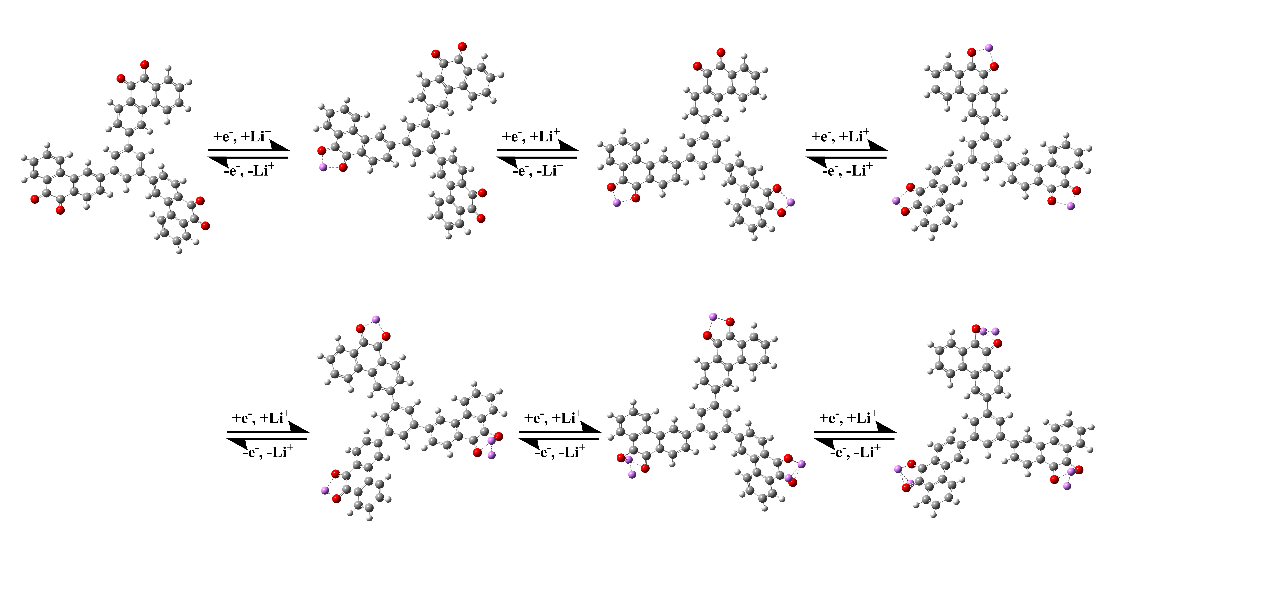


**Figure S16.** the proposed electrochemical redox pathway of TPQB.

**Figure S17.** GCD curves of Ketjen black in 20 cycles.

The TPQB cathodes contain 60 wt.% of active materials, 30 wt.% of KB and 10 wt.% PVDF. The capacities were based on the active materials and the capacity contribution of KB in the electrodes was subtracted. Figure S15 shows that capacity contributions of KB. After cycling stabilization, the capacity is about 25 mAh g^-1^. The calculation formula of the actual capacity of active materials was consistent with above.


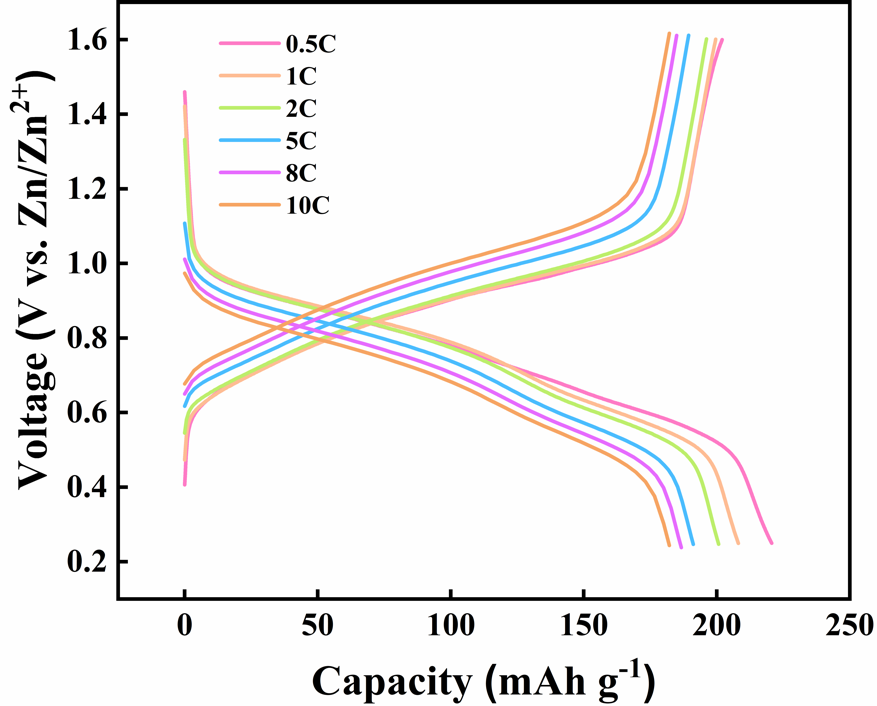


**Figure S18.** GCD curves of TPQB electrode at different current densities.


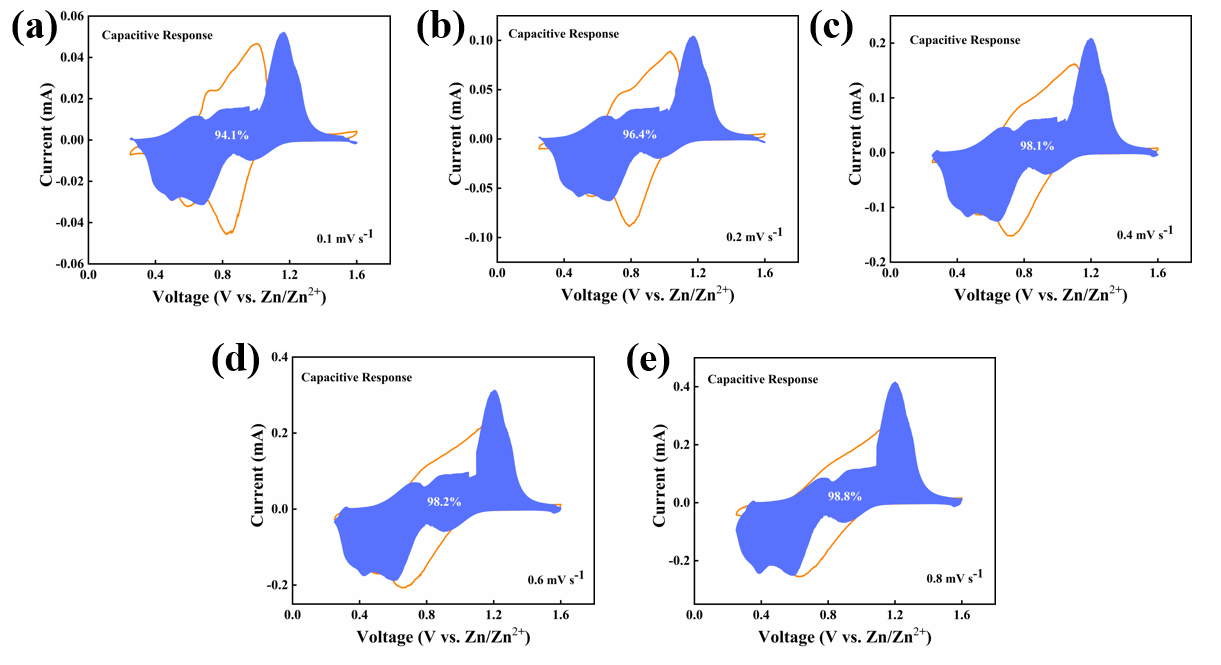


**Figure S19.** CV curves of TPQB cathode and corresponding capacitive contributions at different scan rates.





**Figure S20.** GITT curves as a function of normalized capacity at 0.1 C and diffusion coefficient.

The ion diffusion coefficients (*D*) was calculated using the same formula as above.The higher Zn^2+^ diffusivity (with an average value of 0.7×10^-10^ cm^2^ s^-1^) indicates the fast reaction kinetics and superior ion transport ability of TPQB.





**Figure S21.** Nyquist plots of TPQB cathode in different cycles.

The kinetics of TPQB cathodes was further analyzed by EIS measurements. The progressive stabilization of *R*_ct_ during cycling, indicative of stabilized electrode-electrolyte interfacial dynamics.

**Table S3.** The performance comparison of TPQB//Zn in this work with other organic electrode materials reported in the literatures.

| **Cathode Materials** | **Theoretical**  **capacity**  **(mAh g^−1^)** | **Initial Discharge capacity (mAh g^-1^)** | **Cycle life, Capacity retention** | **Refs.** |
| --- | --- | --- | --- | --- |
| PQ-Δ//Zn | 260.2 | 210 | 500^th^, 99.9%  150 mA g^-1^ | *J. Am. Chem. Soc.* **2020**, *142*, 2541−2548. |
| PQ@AC//Zn ^a)^ | 257.7 | 161 | 36000^th^, 96.3%  5 A g^-1^ | *ACS Appl. Mater. Interfaces* **2021**, *13*, 58818−58826. |
| PQ//Zn | 257.7 | 111 | - | *Sci. Adv.* **2018**, *4*, eaao1761. |
| **TPQB//Zn** | **231.0** | **225.8** | **6000^th^, 93.2%**  **at 5C (1.15 A g^-1^)** | **This work** |

^a)^ Due to the introduction of plentiful activated carbon as host, the specific capacity of composite electrode is great reduced.
